# Endothelial-derived microvesicles promote pro-migratory cross-talk with smooth muscle cells by a mechanism requiring tissue factor and PAR2 activation

**DOI:** 10.3389/fcvm.2024.1365008

**Published:** 2024-06-20

**Authors:** Sophie J. Featherby, Camille Ettelaie

**Affiliations:** Biomedical Section, Hull-York Medical School, Hull, United Kingdom

**Keywords:** tissue factor, smooth muscle cells, endothelial cells, cell migration, microvesicles, filamin A, protease activated receptor-2, serine phosphorylation

## Abstract

**Introduction:**

Microvesicles (MV) released by endothelial cells (EC) following injury or inflammation contain tissue factor (TF) and mediate communication with the underlying smooth muscle cells (SMC). Ser253-phosphorylated TF co-localizes with filamin A at the leading edge of migrating SMC. In this study, the influence of endothelial-derived TF-MV, on human coronary artery SMC (HCASMC) migration was examined.

**Methods and Results:**

MV derived from human coronary artery EC (HCAEC) expressing TF_Wt_ accelerated HCASMC migration, but was lower with cytoplasmic domain-deleted TF. Furthermore, incubation with TF_Asp253_-MV, or expression of TF_Asp253_ in HCASMC, reduced cell migration. Blocking TF-factor VIIa (TF-fVIIa) procoagulant/protease activity, or inhibiting PAR2 signaling on HCASMC, abolished the accelerated migration. Incubation with fVIIa alone increased HCASMC migration, but was significantly enhanced on supplementation with TF. Neither recombinant TF alone, factor Xa, nor PAR2-activating peptide (SLIGKV) influenced cell migration. In other experiments, HCASMC were transfected with peptides corresponding to the cytoplasmic domain of TF prior to stimulation with TF-fVIIa. Cell migration was suppressed only when the peptides were phosphorylated at position of Ser253. Expression of mutant forms of filamin A in HCASMC indicated that the enhancement of migration by TF but not by PDGF-BB, was dependent on the presence of repeat-24 within filamin A. Incubation of HCASMC with TF_Wt_-MV significantly reduced the levels of Smoothelin-B protein, and upregulated *FAK* expression.

**Discussion:**

In conclusion, Ser253-phosphorylated TF and fVIIa released as MV-cargo by EC, act in conjunction with PAR2 on SMC to promote migration and may be crucial for normal arterial homeostasis as well as, during development of vascular disease.

## 1 Introduction

Vascular wall remodeling is an active and adaptable process that occurs in response to injury, inflammation and stress, in order to maintain the vascular integrity and appropriate blood flow. The regulation of these processes involves a vast number of factors including cytokines, inflammatory molecules, cell-cell interaction, extracellular matrix, extracellular vesicles as well as physical influences including sheer stress and external pressure; A satisfactory review of these influences is beyond the scope of this study. Inappropriate signals contributed from these factors can result in hypertrophic, hypotrophic and also eutrophic remodeling of the blood vessel leading to multiple vascular disease conditions (1–6). Blood vessels are primarily comprised of endothelial cells (EC) and vascular smooth muscle cells (SMCs) and the interactions between these cell types is essential in the regulation of vascular tone, the response to injury, and contributes to vascular remodeling. Among the mechanisms of cross-talk involved, extracellular vesicles have emerged as important paracrine messengers (1, 6–10). The bidirectional exchanges of cell-derived microvesicles (MV) contribute to the vascular homeostasis and have been identified as major contributors to vascular abnormalities associated with a number of chronic conditions (11–15). Procoagulant microvesicles are a class of vesicles which are often released in response to injury, trauma, cytokines, infection and other inflammatory mediators (8). Among the pro-inflammatory mediators, the expression and release of tissue factor (TF) has been implicated as one that influences cells through coagulation-dependent and coagulation-independent mechanisms (16, 17). The expression of TF has been associated with phenotypic changes in SMCs (18). Such transformation from contractile to synthetic phenotype is associated with both homeostatic repair processes, and progression of vascular deformities (14). TF has been found within hyperplastic intima of the vasculature from atherosclerotic hearts, associated with the non-lipid rich plaques (19). Moreover, TF is reported to contribute to the progress of coronary artery disease (20, 21). Although under normal circumstances EC do not express TF, stimulation with inflammatory cytokines, endotoxins, hypoxia and oxidative stress, can induce the expression and release of TF as procoagulant microvesicles (22–25). In addition to the above, TF is exposed to the bloodstream through injury and trauma to the vasculature and may be released by other cells including macrophages or cancer cells. Microvesicles act as carriers for TF and factor VIIa (fVIIa), and this cargo may be recycled by EC (26, 27). The resultant endothelial-derived microvesicles may therefore contain both endogenous and recycled TF and fVIIa (28), and since TF-MV have been shown to accumulate within the sub-endothelial regions (29), these may come into contact with SMCs (30–34). Therefore any interaction with underlying cells may in turn activate protease activated receptors (PAR), as well as participating in PAR-independent TF-mediated signals resulting in various outcomes as reviewed previously (25). The contribution of PAR2 to phenotypic alterations in SMCs, and the promotion of SMC migration has also been reported (33, 34). Furthermore, the ability of extracellular vesicles as transporters of miRNA between endothelial and SMCs has been reported (35).

The involvement of actin-binding protein filamin A in vascular repair, remodeling and dysfunction has been well documented (36–38), and the contribution to SMC migration has been studied previously (39–42). TF has been shown to co-localize with filamin A at the leading edge of lamellipodia in migrating SMC and epithelial cells (19, 39) and the interaction of TF with filamin A was demonstrated over two decades ago (43, 44). This interaction was shown to be enhanced following the phosphorylation of the cytoplasmic domain of TF (43, 44). We recently showed that the interaction with filamin A was specifically required the phosphorylation of serine 253 and did not include the phosphorylation of serine 258 (45). In this study, using mutants of TF, peptides corresponding to the cytoplasmic domain of TF, and various antibodies and coagulation factors, we examined the contributions of PAR2 activation and Ser253-phosphorylation within TF, on HCASMC migration. In addition, by expressing mutants of filamin A lacking selected repeat domains in HCASMC, the contribution of the filamin A to TF-induced HCASMC migration was further examined.

## 2 Material and methods

### 2.1 Culture of coronary artery endothelial cell and smooth muscle cell

Human coronary artery endothelial cells (HCAEC) were obtained from PromoCell (Heidelberg, Germany) isolated from left and right main coronary arteries, circumflex and anterior coronary arteries and were CD31 and Dil-Ac-LDL uptake positive. The cells were cultured in EC-MV media containing 5% (v/v) FCS and growth supplements (PromoCell) and remained CD31 positive. Human coronary artery smooth muscle cells (HCASMC) were also isolated and provided by PromoCell as smooth muscle *α*-actin positive, CD31 negative cells. The cells were cultured in SMC-MV2 media containing 5% (v/v) FCS and growth supplements (PromoCell) and were tested for the expression of smoothelin-B which is a marker associated with contractile SMCs from muscular arteries (see discussion for details). Both primary cell types were guaranteed for >15 divisions and were used at around 9 divisions. ECV304 cell line was cultured in M199 medium containing 10% (v/v) FCS.

### 2.2 Preparation of TF and filamin A mutants

The sequence for full length filamin A was cloned into pcDNA 3 c-myc plasmid and stop codons were engineered into positions Gly2370, Gly2467 and Ala2595 in order to delete the repeat units 22–24, 23–24, or 24 alone. The successful mutations were verified by DNA sequencing (MWG-eurofins, Wolverhampton, UK). The pCMV6-Ac-TF-tGFP plasmid for the expression of full-length wild-type human TF (TF_Wt_-tGFP) was obtained from OriGene (Rockville, USA) and mutations were prepared as previously described (46) to express proteins with Ser253Asp (TF_Asp253_-tGFP), truncated at Ser241 (TF*_Δ_*_CT_-tGFP), or tGFP alone.

### 2.3 Transfection of HCAEC and induction and isolation of MV

Human coronary artery endothelial cells (HCAEC) were used throughout the investigation because these cells are the closest representatives to the aims of this investigation. In contrast to monocytes or tumor cells, these cells do not either constitutively express TF and/or release large amounts of microvesicles spontaneously. Furthermore, unlike tumor cell derived microvesicles, the resultant microvesicles do not contain other material that alter the behavior of vascular cells (e.g., pro-angiogenic mediators). HCAEC have also been shown to express PAR2 on the cell surface (47). HCAEC (5 × 10^5^) and also in some experiments ECV304 cells, were transfected with 0.5 µg of pCMV6-Ac-TF-tGFP plasmid variants using TransIT-2020 (Geneflow, Litchfield, UK) according to the manufacturer's instructions and permitted to express the proteins for 48 h prior to use. The expression of TF was previously confirmed by measuring TF mRNA and measuring the total and cell-surface antigen levels by western blot and flow cytometry (46, 47). All cell samples, including the non-transfected cells, were then transferred and adapted to serum-free medium for 1 h. The release of TF-containing microvesicles was induced by incubation with PAR2 activating peptide (PAR2-AP); SLIGKV (20 µM; Sigma Chemical Company Ltd, Poole, UK). Following 80 min incubation with PAR2-AP, the conditioned media were cleared of any cell debris by centrifuging for 10 min at 2,500 g on a microcentrifuge. The samples (1 ml aliquots) were then placed in 11 × 34 mm polycarbonate centrifuge tubes (Beckman Coulter, High Wycombe, UK), and the cell-derived microvesicles were sedimented at 100,000 g on a TL-100 ultracentrifuge at 20°C, using a TLA 100.2 rotor (Beckman) for 1 h, as described before (46, 48). Sedimented microvesicles were washed with PBS. The pellet was resuspended in pre-filtered (0.1 μm) PBS (200 µl), divided into batches, and frozen at −80°C or used immediately. The specificity of the agonist in the incorporation and release of TF was previously demonstrated and compared to the ability of factor Xa to promote the release of TF-containing microvesicles (46). Additionally we showed that freezing of the microvesicles did not alter the microvesicle size or number as determined by nanoparticle tracking analysis (48), the TF antigen levels (46, 47) or the TF associated thrombin generation and factor Xa-generation. Furthermore, pre-incubation of the microvesicles with antibodies against TF, or immune-purification of the microvesicles using TF antibodies did not alter the size distribution of the microvesicles (Supplementary Figure S1), or the total TF content (48).

Previously, the microvesicles released following PAR2-AP activation were compared to those released following activation with TNF*α* (10 ng/ml) or IL-1β (10 ng/ml) (46) and microvesicles released from THP-1 cells, isolated peripheral blood mononuclear cells (PBMC), or HCASMC, in parallel (46, 47). The properties of TF-containing microvesicles from these various sources prepared by different procedures were established previously; in particular the numbers and size distributions of the isolated microvesicles were determined by nanoparticle tracking analysis (NTA) using Nano-Sight LM10 and NTA software (NanoSight Ltd, Amesbury, UK). The Nanosight instrument was calibrated using FluoSpheres® carboxylate-modified microspheres with diameters of 0.1 μm and 1.0 μm (Invitrogen). Microvesicle samples were diluted 1:5 in 0.1 μm-filtered PBS and analyzed using the NanoSight LM10 by tracking particles over 60 s using a camera level of 12 and shutter speed of 21.26 frames/s. A typical NTA trace for microvesicles is included in Supplementary Figure S1. Additionally, size and density of the TF-containing microvesicles were established previously by density gradient centrifugation (48) and the phospholipid content assessed by thin layer chromatography (46). Importantly, the density of the released microvesicles was measured by the concentration of phosphatidylserine, which was determined using the Zymuphen MP-assay kit (Hyphen BioMed/ Quadratech, Epsom, UK) against the standards provided with the kit. This function is the most relevant to this study. The TF content of the microvesicles was also measured by flow cytometry (46–48), TF-specific ELISA (Quantikine, R&D Systems, Abingdon, UK) and western blot (49). Although on the whole the resultant microvesicles were comparable in TF content and microvesicle identity, repeated studies in our laboratory has indicated the release of more consistent TF-containing microvesicles following activation by PAR2-AP and therefore this procedure has been adopted by our laboratory as the most consistent and reproducible procedure for preparing TF-MV (45–49). Previous examination of the microvesicles for exosome markers Tsg101 and CD9 by western blot showed that exosomes were not present, which was likely due to the short incubation times (26, 46, 47). Similarly, exosome marker antibodies (anti-Tsg101 or CD9 antibodies) were not capable pulling down TF-microvesicles (48).

### 2.4 Transfection of HCASMC

HCASMC were transfected with the pcDNA 3 c-myc constructs using TransIT 2020 reagent to express the wild type or mutant forms of filamin A, lacking repeats domains (22–24, 23–24 and 24 alone). The cells were permitted to express the proteins for 48 h and then employed in migration assays as below. The expression of the proteins was confirmed using a biotin-conjugated mouse anti-c-myc antibody (Miltenyi Biotech Ltd., Woking, UK), probed with HRP-conjugated streptavidin (Cytivia Life Science/ Fisher Scientific UK Ltd., Loughborough, UK) and developed using TMB stabilized substrate (Promega Corp., Southampton, UK). Parallel sets of samples were also examined using a rabbit anti-filamin A antibody (EP2405Y) (Epitomics Inc, Burlingame, CA, USA). The cells were then probed with goat anti-rabbit-alkaline phosphatase antibody (Santa Cruz Biotechnology) and developed using Western Blue substrate (Promega). In some experiments, HCASMC were transfected using TransIT-2020, to express TF-tGFP variants as described for the EC. In other experiments, HCASMC were transfected with a set of peptides corresponding to the cytoplasmic domain of TF with different serine-phosphorylation patterns (50), as stated in the results section. Briefly, the peptides (0.5 µg) were diluted in PBS (100 µl) and Chariot reagent (6 µl; Active Motif, La Hulpe, Belgium) was also diluted in PBS. The two reagents were then mixed and incubated for 30 min. HCASMC were adapted to serum-free medium (400 µl) for 1 h before and the Chariot-peptide mix (200 µl) was added to the cells. The cells were incubated for a further 60 min, the medium replaced and used in the experiments.

### 2.5 Treatment of MV and HCASMC with antibodies, proteins and other reagents

In some experiments, the isolated MV were pre-incubated for 1 h, with a mouse anti-human-TF antibody, 10H10 (20 µg/ml; BD Bioscience, Wokingham, UK) capable of blocking TF signaling, a mouse anti-human-TF antibody, HTF-1 (20 µg/ml; eBioscience/Thermo Scientific, Warrington, UK) to block TF-fVIIa protease/procoagulant activity, an inhibitory polyclonal rabbit anti-human fVIIa antibody (10 µg/ml; Abcam, Cambridge, UK) or the respective control isotype IgG antibodies (20 µg/ml; New England Biolabs, Hitchin, UK). The MV were then added to the lower chamber in migration assays as below. Alternatively, HCASMC were treated with a mouse anti-human PAR2 antibody, SAM11 (20 µg/ml; Santa Cruz Biotechnology, Heidelberg, Germany), capable of blocking PAR2 signaling, or PAR2-agonsit peptide (PAR2-AP) to induce PAR2 signaling. SAM11 antibody was added with the HCASMC in the upper chamber at the start of the experiment and PAR2-AP was added to the lower chamber. PDGF-BB and PDGF-AA (10 ng/ml) were also added to sets of cells, in the bottom chamber as positive and negative controls, respectively. Additionally, to examine the influence of TF alone, and the contributions of other factors, HCASMC were stimulated with combination of recombinant Innovin TF (0–260 pg/ml; Dade Behring, Deerfield, USA), fVIIa (3 nM; Enzyme Research Lab., Swansea, UK), fXa (6 nM; Enzyme Research Lab.) and tissue factor pathway inhibitor (TFPI; 1 nM; American Diagnostica/Axis-Shield Ltd, Dundee, UK). To examine the contribution of activation of FAK, HCASMC were pre-incubated with either FAK inhibitor-14 (0–100 μM; 1,2,4,5-benzenetetraamine tetrahydrochloride; Sigma Chemical Company Ltd, Poole, UK) to inhibit FAK autophosphorylation, or AG82 {2-[(3,4,5-trihydroxyphenyl)methylene]-propanedinitrile}; (0–10 μM; Calbiochem/Merck-Millipore, Gillingham, UK) that prevents receptor-kinase mediated p125FAK tyrosine phosphorylation. Other sets of cells were pre-incubated with calyculin-A (1 nM) in order to prevent TF de-phosphorylation.

### 2.6 HCASMC migration assays

We have previously shown that exogenous-TF can become incorporated into the recipient cell membrane (26, 28, 47, 51). The HCASMC migration assay relies on the ability of cells to traverse a membrane to the lower chamber using Boyden chambers (8 μm pore size) (VWR International Ltd., Leicestershire, UK). For each assay, identical number of cells (approximately 3 × 10^4^ in 250 μl of complete SMC-MV2 media) were placed in the upper chamber. If required, the cells were supplemented with antibodies directed towards the cellular receptors (e.g., SAM11), or other reagents. Complete media (250 μl) was placed in the lower chamber containing the test reagent as described above and indicated in the results section. The chambers were incubated at 37°C for 18 h. The cells were then fixed with glutaraldehyde (3% v/v), washed with PBS and then, the cells on the upper side of the chamber were scraped off. To quantify the number of migrating cells, the scraped chambers were stained with crystal violet solution (Active Motif) for 30 min, washed and 10 fields of view were photographed. The cell-associated crystal violet was then released from the cells by incubation with 1% (w/v) SDS (200 μl) for 20 min. The absorptions were then measured at 595 nm and migrated cell numbers were determined from a standard curve prepared using HCASMC. In some experiments, the chambers were washed and stained with haematoxylin for visualization and 10 fields of view were counted manually or subsequently stained with crystal violet as above. For clearer demonstration of HCASMC migration, alternate images have been presented in the results section displaying cells stained with crystal violet at ×4, or at ×10 magnification.

### 2.7 RNA isolation, RT-PCR and western blot analysis

Total RNA was isolated using the Monarch total RNA extraction kit (New England Biolabs, Hitchin, UK) from 2 × 10^5^ cells and 100 ng of total RNA was used for each reaction. The relative amounts of *FAK* mRNA was determined using the primer (forward 5′-AATCCTGGAGGAAGAGAAGGC-3′; reverse 5′-TGTTGCTGTCGGATTAGACGC-3′) and semi-qualitative analysis carried out in comparison to β*-actin* (forward 5′-CCAGAGCAAGAGAGGCATCC-3′; reverse 5′-CTGTGGTGGTGAAGCTGTAG-3′). One-step RT-PCR reaction was carried out with 100 ng of isolated total RNA using Ready-To-Go RT-PCR Beads (Amersham Pharmacia Biotech, Inc., Giles, UK) for 30 cycles for *FAK* or 18 cycles for β*-actin*. RT-PCR products were visualized on a 2% (w/v) agarose gel, stained with SYBR Green I (FMC BioProducts, Rockland, USA).

Western blot analysis was carried out to determine the amount of Smoothelin-B protein in the HCASMC. Cells (10^5^) were lysed in loading buffer and electrophoresis was carried out on a denaturing 8% (w/v) polyacrylamide gel, transferred to a nitrocellulose membrane and blocked with Tris-buffered saline Tween 0.01% (v/v) (TBST) pH 8. The membranes were probed overnight with a rabbit anti-human Smoothlein antibody (diluted 1:3,000 v/v; Abbexa, Cambridge, UK), or a goat anti-human GAPDH (V18) antibody (diluted 1:3,000 v/v; Santa Cruz Biotechnology, Heidelberg, Germany). The membranes were then washed and developed with goat anti-rabbit, or donkey anti-goat alkaline phosphatase-conjugated antibodies (Santa Cruz), diluted 1:3,000 (v/v), and visualized using the Western Blue stabilized alkaline phosphatase-substrate (Promega).

### 2.8 Statistical analysis

Presented data include the calculated mean values ± the calculated standard error of the mean, and the number of experiments is stated in each column. Statistical analysis was carried out using the GraphPad Prism version 9.0 (GraphPad Software, Boston, Massachusetts USA). Significance was determined using one-way ANOVA (analysis of variance) and Tukey's honesty significance test.

## 3 Results

### 3.1 TF-containing MV derived from HCAEC, and recombinant TF-fVIIa enhance HCASMC migration

**Figure 1:**
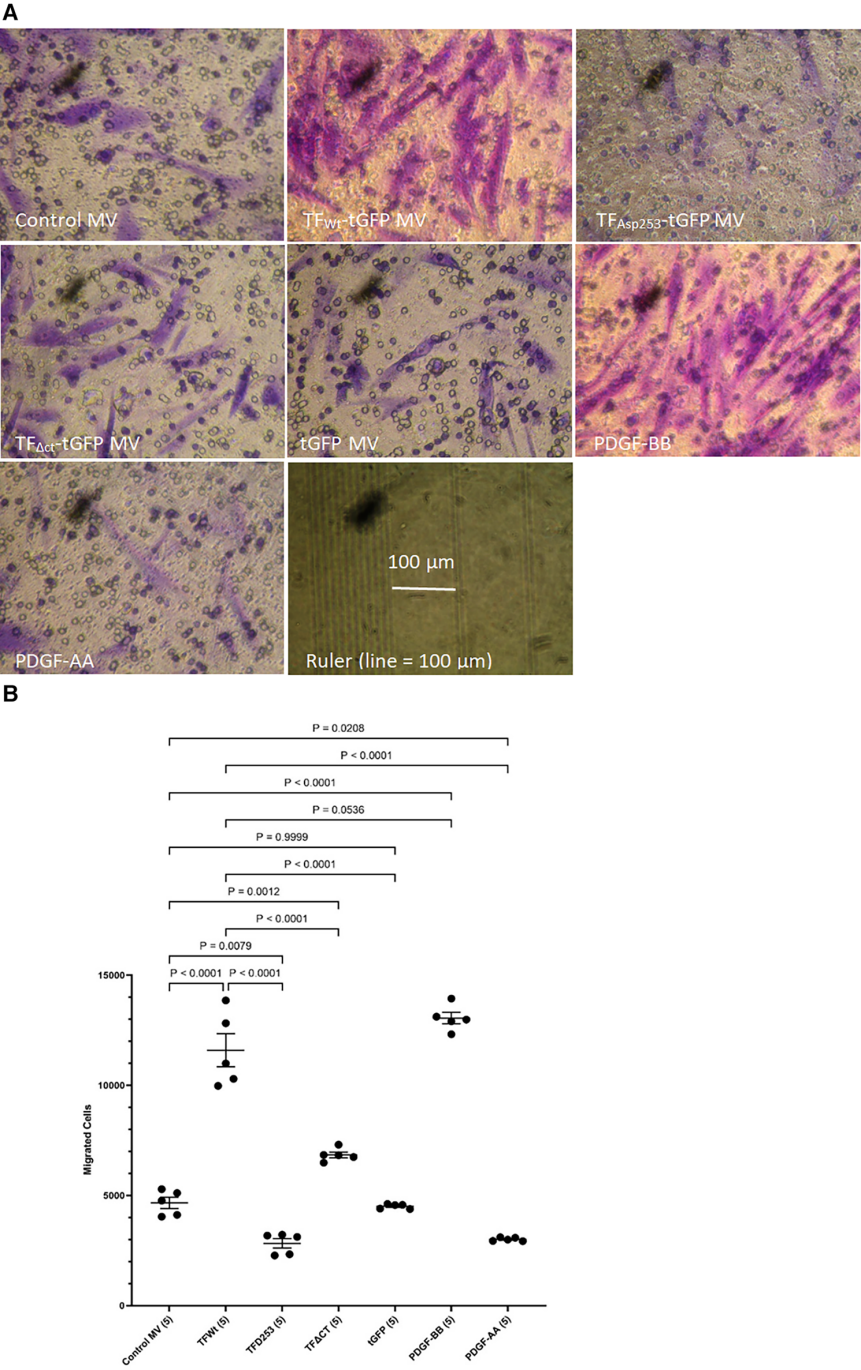
Examination of HCASMC migration in response to TF-containing MV. MV were prepared from HCAEC (5 × 10^5^) transfected to express TF_Wt_-tGFP, TF_Asp253_-tGFP, TF_ΔCT_-tGFP or tGFP alone. HCASMC (3 × 10^4^ in 250 μl of media) were placed in the upper chamber of Boyden chambers. Complete media (250 μl), containing the isolated MV, was placed in the lower chamber and incubated at 37°C for 18 h. Additional sets of cells were incubated with PDGF-BB (10 ng/ml) or PDGF-AA (10 ng/ml) as positive and negative controls as well as non-treated samples. After incubation, the cells were fixed, washed with PBS and the cells on the upper side of the chamber were scraped off. (**A**) The cells were stained with crystal violet and photographed and (**B**) the numbers of migrating cells were determined by eluting the crystal violet and measuring the absorptions at 595 nm. Presented data include the calculated mean values ± the calculated standard error of the mean, from 5 experiments.

HCASMC were incubated with MV isolated from HCAEC expressing TF_Wt_-tGFP, TF_Asp253_-tGFP, TF_*Δ*CT_-tGFP, tGFP alone, or from non-transfected cells (control MV). The incorporation of TF-tGFP into the cells and interaction with filamin A (probed with EP2405Y antibody) was monitored qualitatively, by confocal microscopy (Supplementary Figure S2). In addition, the association of TF-tGFP with the c-terminal of filamin A was confirmed *in situ* by proximity ligation assay as before (28, 45), and using a rabbit monoclonal antibody against the c-terminal of filamin A (EP2405Y), in conjunction with first a mouse monoclonal antibody to TF (10H10) and also, with a mouse monoclonal antibody to tGFP protein (2HB, OriGene) (Supplementary Figure S3). The MV were isolated using an established ultracentrifugation procedure, confirmed and quantified as before (48). Selected experiments were repeated using MV derived from transfected ECV304 line. These cells do not express TF under normal conditions but the transfected cells express the recombinant protein efficiently, and the cells release large quantities of MV on PAR2 activation. Typically, the yield of MV ranged between 0.25–0.31 nM and the concentration of TF in the MV from cells that expressed TF-tGFP was between 1 and 3 ng/ml. For the HCASMC migration experiments, 0.5 pg/ml (or equivalent amount of control MV) was placed in the bottom chamber of the Boyden chamber. Additional sets of HCASMC were supplemented with PDGF-BB or PDGF-AA which was added to the bottom chamber, as positive and negative controls, as well as sets of non-treated sample. The reagents were placed in the bottom chamber and the rate of cell migration across the Boyden chamber measured. Incubation of HCASMC with MV containing TF_Wt_-tGFP resulted in increased cell migration compared to cells expressing either tGFP alone, or the control MV. The increases were comparable to but lower than those obtained using the positive control (PDGF-BB) (Figures 1A,B and Supplementary Figure S4). However, the rate of migration was lower with TF_ΔCT_-tGFP indicating that the cytoplasmic domain of TF is essential for full effectiveness of the signal. Interestingly, incubation of cells with MV containing TF_Asp253_-tGFP resulted in reduced cell migration to levels comparable to the negative control (PDGF-AA).

**Figure 2:**
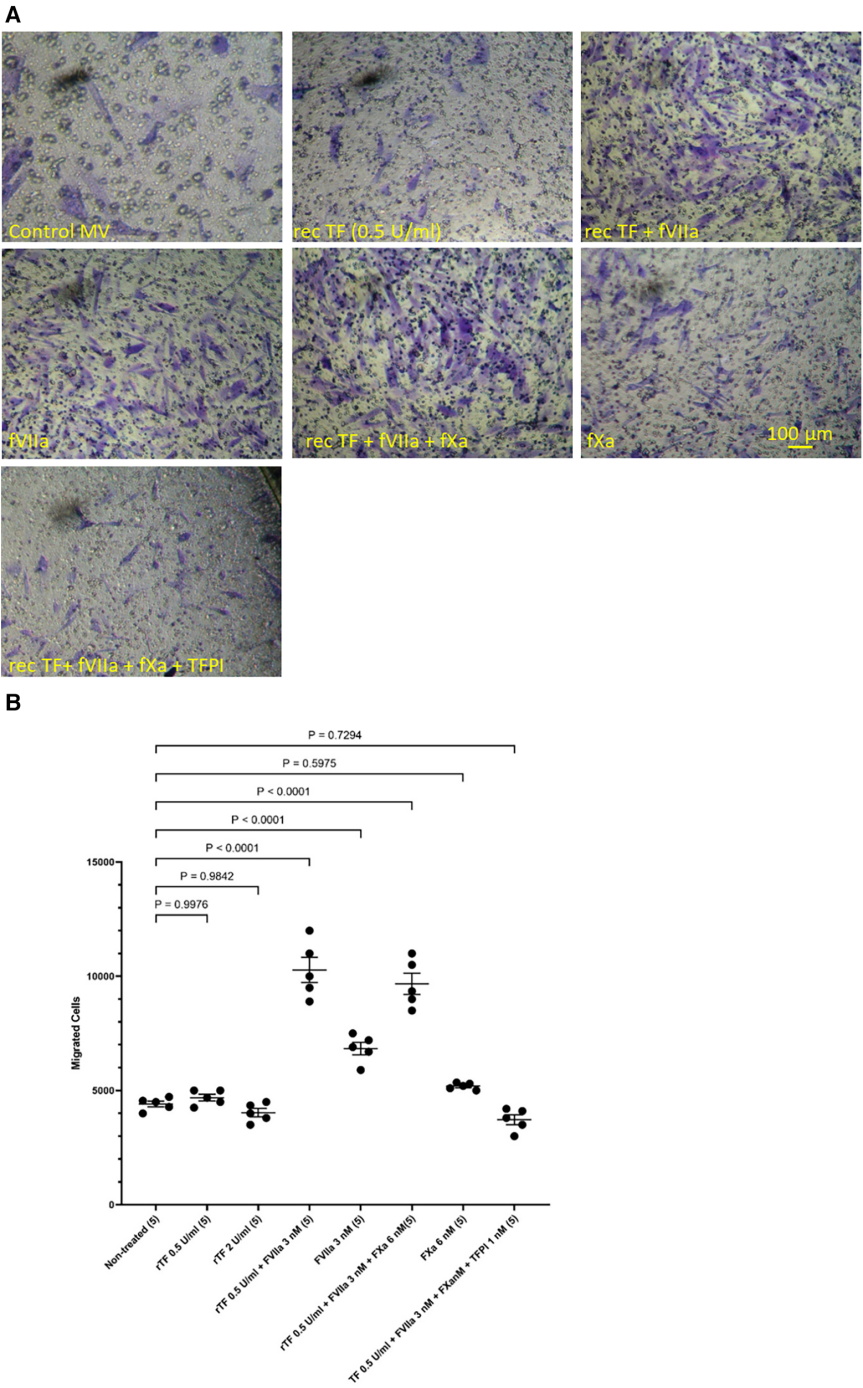
Examination of HCASMC migration in response to TF, fVIIa, fXa and TFPI. HCASMC (3 × 10^4^ in 250 μl of media) were stimulated with combinations of recombinant Innovin TF (0–260 pg/ml), fVIIa (3 nM), fXa (6 nM) and TFPI (1 nM) and incubated at 37°C for 18 h. The cells were then fixed, washed and the cells on the upper side of the chamber were scraped off. (**A**) The cells were stained with crystal violet and photographed and (**B**) the numbers of migrating cells were determined by eluting the crystal violet and measuring the absorptions at 595 nm. Presented data include the calculated mean values ± the calculated standard error of the mean, from 5 experiments.

**Figure 3:**
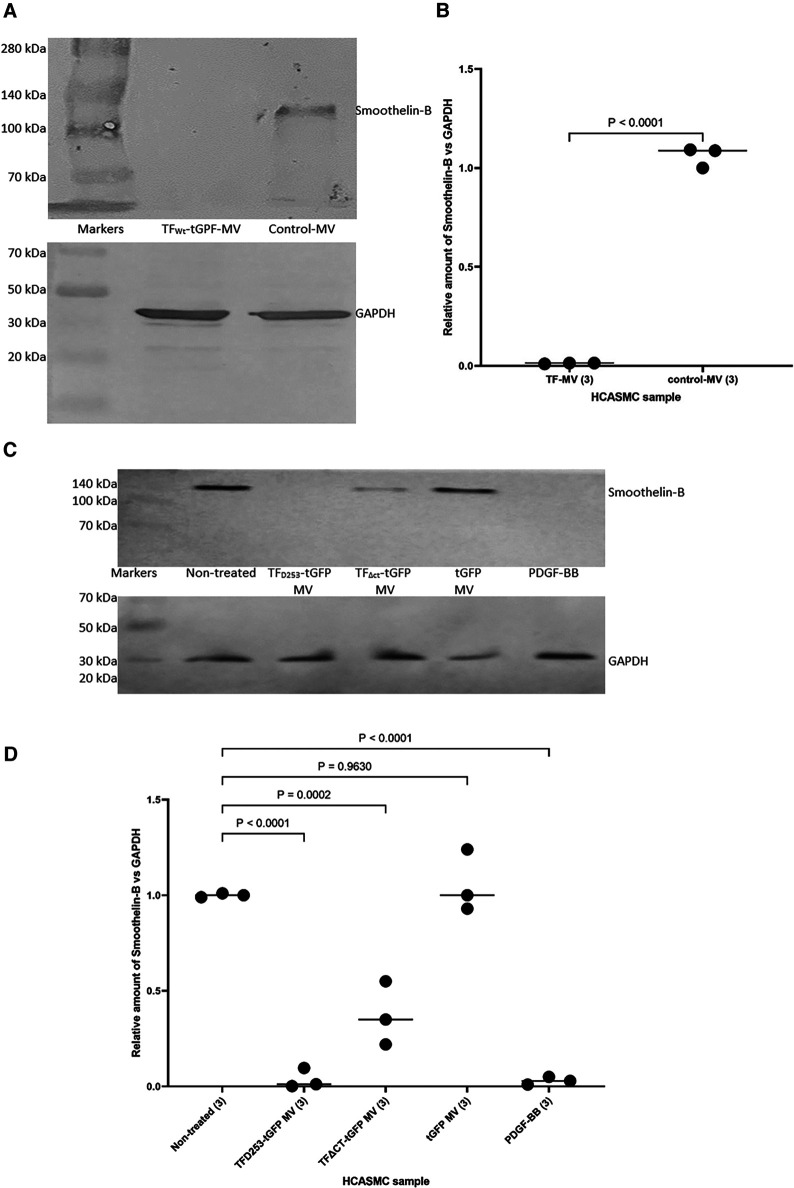
The influence of TF-fVIIa on the expression of Smoothelin-B in HCASMC. (**A**) HCASMC (10^5^) were treated with MV prepared from HCAEC (5 × 10^5^) transfected to express TF_Wt_-tGFP, or non-transfected cells. The cells were lysed and the proteins separated by 8% (w/v) polyacrylamide electrophoresis, transferred to nitrocellulose membranes, blocked and probed with a rabbit anti-human Smoothlein antibody (diluted 1:3,000 v/v), or a goat anti-human GAPDH (V18) antibody (diluted 1:3,000 v/v). The membranes were then washed and developed with goat anti-rabbit, or donkey anti-goat alkaline phosphatase-conjugated antibodies diluted 1:3,000 (v/v), and visualized using the Western Blue stabilized alkaline phosphatase-substrate. (**B**) The density of the Smoothelin-B bands was determined and expressed as a ratio of that of GAPDH in each sample. Presented data include the calculated mean values ± the calculated standard error of the mean, from 3 experiments. (**C**) Similarly, HCASMC were treated with MV containing TF_Asp253_-tGFP, TF_ΔCT_-tGFP, tGFP or incubated with PDGF-BB. (**D**) The expression of Smoothelin-B was then examined as above and the density of the Smoothelin-B bands was expressed as a ratio of that of GAPDH in each sample. Presented data include the calculated mean values ± the calculated standard error of the mean, from 3 experiments.

In addition, HCASMC were incubated with combinations of recombinant TF, fVIIa, fXa and TFPI. Incubation of cells with the combination of TF and fVIIa resulted in increased cell migration but was not significantly altered by supplementation with fXa (Figures 2A,B). In contrast, neither concentration of recombinant TF was capable of promoting HCASMC migration in the absence of fVIIa. Furthermore, stimulation with fVIIa alone was less effective in inducing HCASMC migration while fXa alone was only marginally effective. In accordance with previous studies, HCASMC expressed endogenous TF but the levels were low in our study which is also in agreement with the levels reported in healthy vessels (52–55). However, neither the zymogen nor activated fVII/VIIa were detected in HCASMC when examined by western blot (not shown), using a previously verified polyclonal anti-fVIIa antibody (56). Moreover, inclusion of recombinant TFPI reduced the induction of migration. In an attempt to examine the state of HCASMC, the expression of the Smoothelin was examined by western blot analysis. Smoothelin-B was shown to be present in resting HCASMC but not following treatment with TF-fVIIa (Figure 3). However, other markers were not examined in this study. Similarly, the expression of Smoothelin-B was suppressed in HCASMC treated with MV containing TF_Asp253_-tGFP, or with PDGF-BB, was partially reduced in cells treated with MV containing TF*_Δ_*_CT_-tGFP but was not altered in cells treated with tGFP-MV (Figures 3C,D).

### 3.2 PAR2 activation is essential for the induction of TF-mediated HCASMC migration

**Figure 4:**
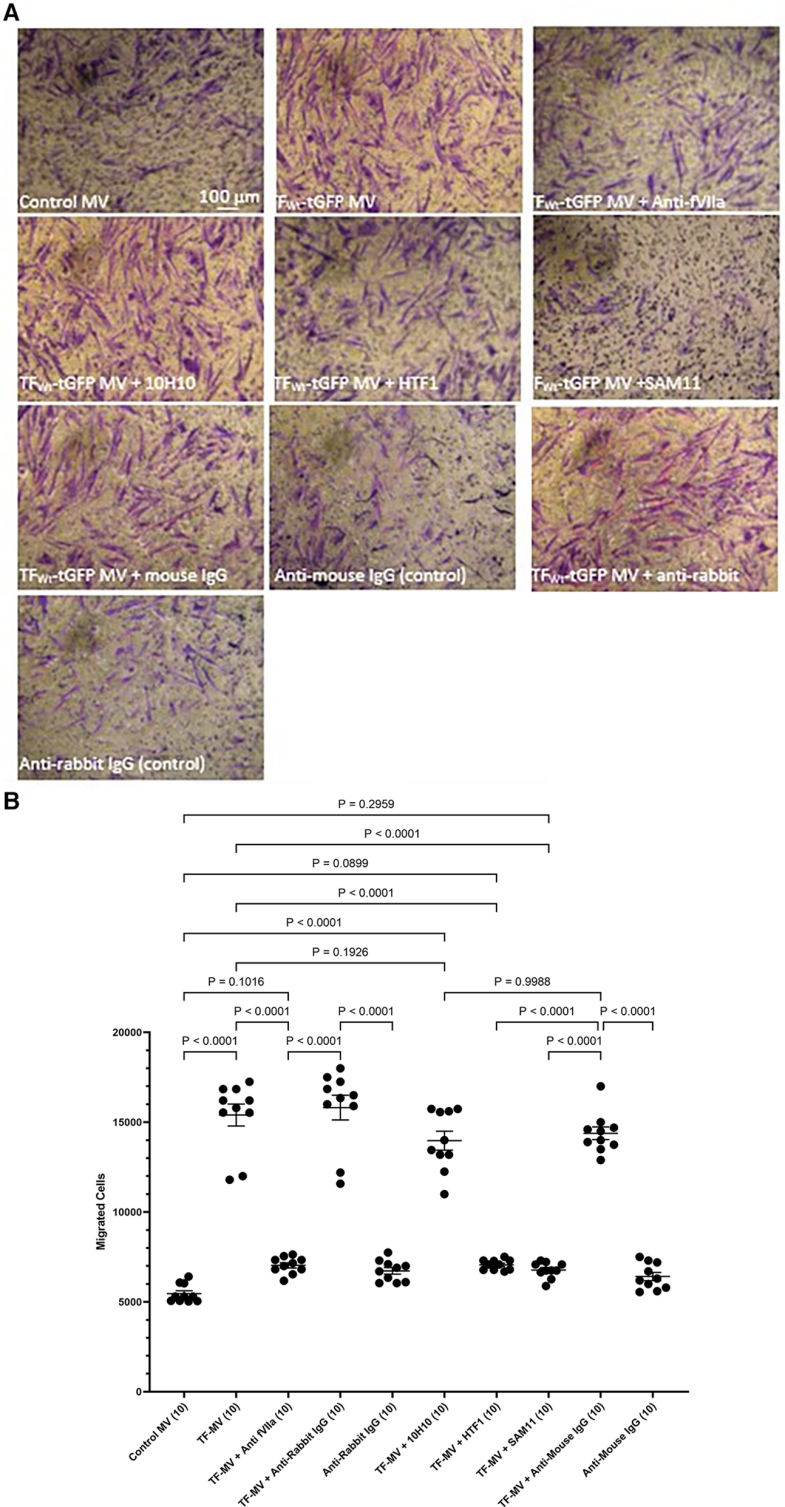
Examination of the pro-migratory mechanism of TF-MV using blocking antibodies. MV were prepared from HCAEC (5 × 10^5^) transfected to express TF_Wt_-tGFP and pre-incubated with mouse anti-human-TF antibodies, 10H10 (20 µg/ml) or HTF1 (20 µg/ml), or an inhibitory polyclonal rabbit anti-human fVIIa antibody (10 µg/ml). Additionally, HCASMC were incubated with an inhibitory mouse anti-human PAR2 antibody, SAM11 (20 µg/ml). HCASMC (3 × 10^4^) were stimulated with MV at 37°C for 18 h. The cells were then fixed, washed and the cells on the upper side of the chamber were scraped off. (**A**) The cells were stained with crystal violet and photographed and (**B**) the numbers of migrating cells were determined by eluting the crystal violet and measuring the absorptions at 595 nm. Presented data include the calculated mean values ± the calculated standard error of the mean, from 5 experiments each examined in duplicate.

To further examine the underlying mechanisms of induction of HCASMC migration by TF, MV were isolated from HCAEC and ECV304 cell line that were transfected to express TF_Wt_-tGFP, and then pre-incubated with an inhibitory polyclonal antibody against fVIIa (56, 57), a monoclonal anti-TF antibody capable inhibiting the procoagulant activity (HTF-1) (58), or a monoclonal antibody that blocks TF-mediated signaling (10H10) (59), before adding the MV to the lower chamber. These antibodies are directed to the extracellular domain of TF and fVIIa also associates with the extracellular domain. Alternatively, the HCASMC were pre-incubated with a monoclonal antibody capable of blocking PAR2 activation and signaling (SAM11). Appropriate isotype antibodies were analyzed alongside according to the species of the antibody used. The inclusion of these antibodies did not alter the cell migration by TF-MV, and the isotypes IgG antibodies showed no influence otherwise (Figure 4). The effectiveness of TF-tGFP-containing MV to induce HCASMC migration was largely abolished on blocking PAR2 signaling by pre-incubation of HCASMC with SAM11 (Figures 4A,B and Supplementary Figure S5). Similarly, neutralization of TF-fVIIa protease activity, by pre-incubation with an inhibitory anti-fVIIa antibody, or incubation with HTF-1 antibody significantly reduced the rate of HCASMC migration while, the blocking of TF signaling using 10H10 antibody was ineffective. Stimulation of HCASMC with the PAR2 activating peptide (PAR2-AP, SLIGKV) or PAR1 activating peptide (PAR1-AP, SFLLRN) did not alter the rate of HCASMC migration, while pre-incubation of HCASMC with a monoclonal antibody to block β1-integrin completely abolished any cellular migration (not shown).

### 3.3 Phosphorylation of Ser253 but not Ser258 is required for the enhanced HCASMC migration

**Figure 5:**
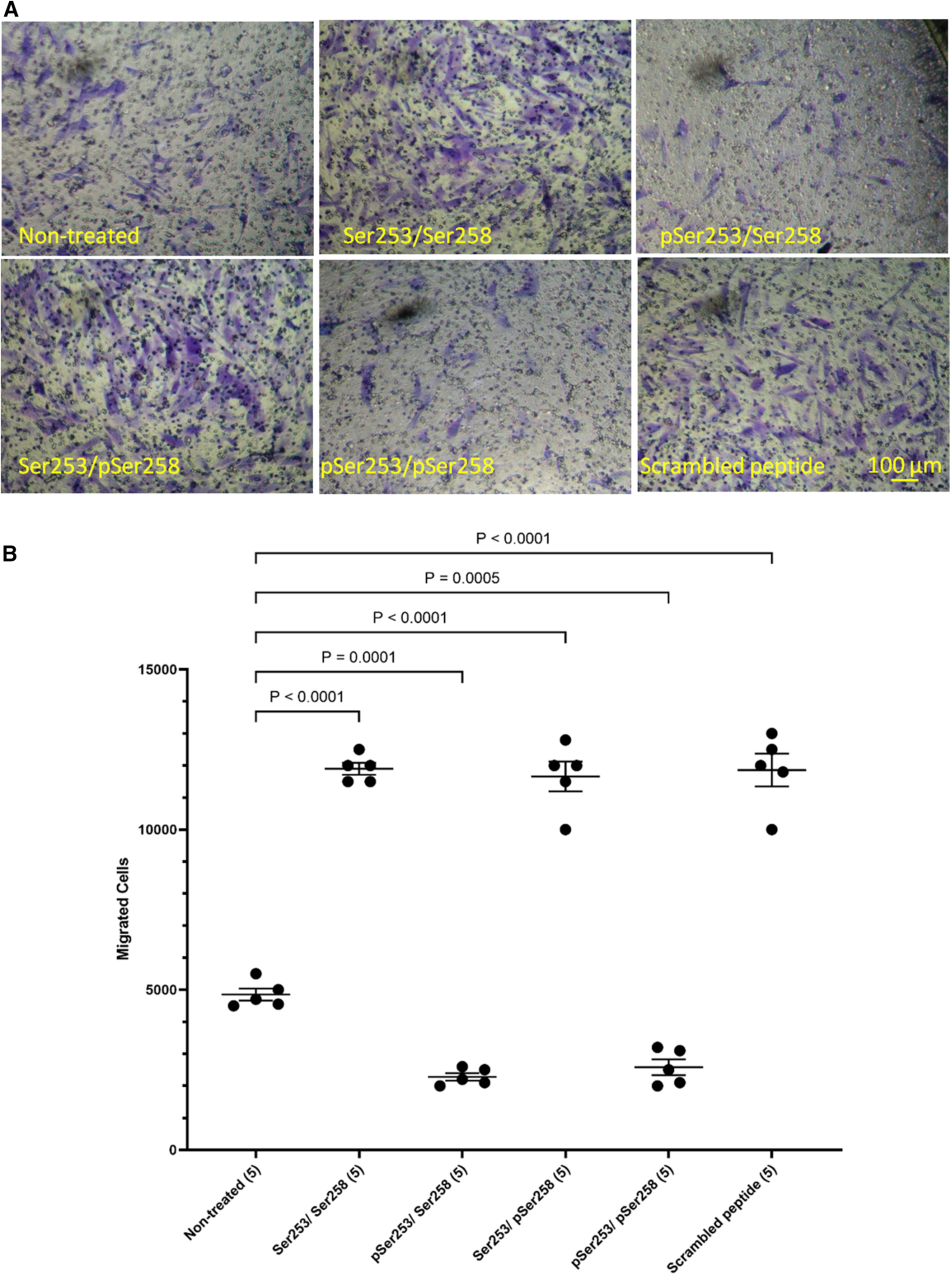
Examination of the influence of TF phosphorylation on HCASMC migration. HCASMC were transfected with a set of peptides corresponding to the cytoplasmic domain of TF with different serine-phosphorylation patterns at the three serine residues. Migration was induced in the transfected HCASMC with TF-MV at 37°C for 18 h. The cells were then fixed, washed and the cells on the upper side of the chamber were scraped off. (**A**) The cells were separately stained with crystal violet and (**B**) the numbers of migrating cells were determined by eluting the crystal violet and measuring the absorptions at 595 nm. Presented data include the calculated mean values ± the calculated standard error of the mean, from 5 experiments.

In order to examine the role of TF phosphorylation, HCASMC were transfected with peptides corresponding to the 18 amino acids of the cytoplasmic domain of TF and the rate of migration examined as above. The transfection of a similar FITC-labelled peptide into the cells was confirmed by flow cytometry which indicated up to 85% transfection rate in both washed and unwashed cells and mean fluorescence intensity of up to 3 orders of magnitude (Supplementary Figure S6). Additionally, transfection of the peptides does not influence cell viability (46, 50). Transfection with either form of the peptide, incorporating a phosphorylated-Ser253 (pSer253 or pSer253/pSer258) resulted in significant reduction in the number of migrating cells in response to TF-fVIIa (Figures 5A,B). In contrast, the peptide incorporating phospho-Ser258 (pSer258) and the non-phosphorylated form were largely ineffective.

### 3.4 Induction of HCASMC migration is mediated through binding to filamin A

**Figure 6:**
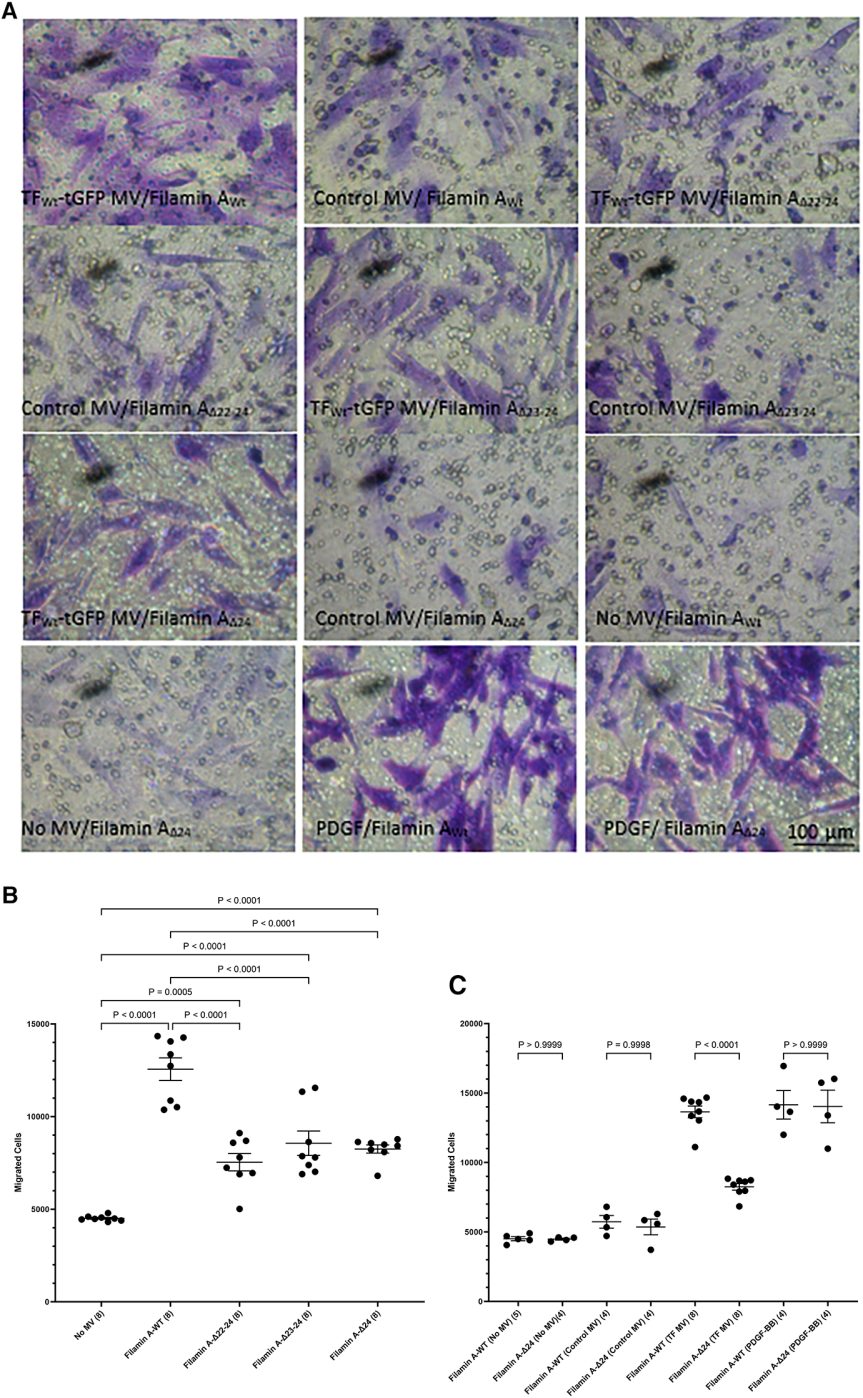
Examination of the requirement for filamin A repeats 22–24 for TF-induced HCASMC migration. HCASMC were transfected with the pcDNA 3 c-myc constructs to express the wild type or mutant forms of filamin A, lacking repeats domains (22–24, 23–24 and 24 alone) and permitted to express the proteins for 48 h. Cells (3 × 10^4^ in 250 μl of media) were placed in the upper chamber of Boyden chambers. Complete media (250 μl), including MV containing TF_Wt_-tGFP, was placed in the lower chambers and incubated at 37°C for 18 h. Additional sets of cells were incubated with PDGF-BB (10 ng/ml) control MV (from non-transfected HCAEC), as well as sets of non-treated sample. The cells were then fixed, washed and the cells on the upper side of the chamber were scraped off. (**A**) The cells were stained with crystal violet and photographed. (**B**) The number of migrating cells were determined in HCASMC, in response to TF_Wt_-tGFP containing MV, and compared to control MV. Presented data include the calculated mean values ± the calculated standard error of the mean, from 4 experiments, each examined in duplicate. (**C**) The migration of HCASMC, expressing wild type filamin A, and filamin A lacking repeat-24, in response to TF_Wt_-tGFP containing MV, control MV, PDGF-BB (10 ng/ml), or without any supplement were determined. The rates of migration were measured by staining with crystal violet which was then eluted and absorptions measured at 595 nm. Presented data include the calculated mean values ± the calculated standard error of the mean, from 8 experiments for the samples treated with TF-MV, and 4 experiments for all other samples, as shown on each column.

Phosphorylation of Ser253 is known to be essential for the interaction of TF with the repeat domain 22–24 within filamin A (43, 45). Additionally, TF is known to colocalize with filamin A at the leading edges of migrating SMCs (19). To further assess the contribution of filamin A to TF-mediated cell migration, HCASMC were transfected to express three truncated forms of filamin A without the repeat domains 22–24, 23–24 and 24 alone. The expression of the filamin A variants (filamin A_Wt_, filamin A_Δ22−24_, filamin A_Δ23−24_ and filamin A*_Δ_*_24_) was confirmed by western blot, probed using an antibody against the c-myc and anti-filamin A (Supplementary Figure S7). Moreover, the lack of any detrimental outcome on cell viability was established by determining the cell numbers in the transfected and control cells. HCASMC migration across Boyden chamber membranes was then stimulated with the HCAEC-derived MV containing TF_Wt_-tGFP, or devoid of TF (control MV). Removal of either set of repeat domains (22–24, 23–24 or 24 alone) resulted in significantly reduced HCASMC migration (Figures 6A,B). Next, whether repeat-24 is required specifically for TF-induced migration, or also affects other/non-specific migration mechanisms/processes, was examined. Expression of either filamin A_Wt_, or filamin A_Δ24_ resulted in similar rates of migration in unstimulated HCASMC, cells stimulated with control MV, or in response to PDGF-BB (10 ng/ml) (Figures 6A,C). These were in contrast to the difference in migration observed on stimulation with TF-MV. Consequently, this study indicates that repeat-24 is likely to participate in TF-specific promotion of migration in HCASMC.

### 3.5 De-phosphorylation of TF at Ser253 permits HCASMC migration

**Figure 7:**
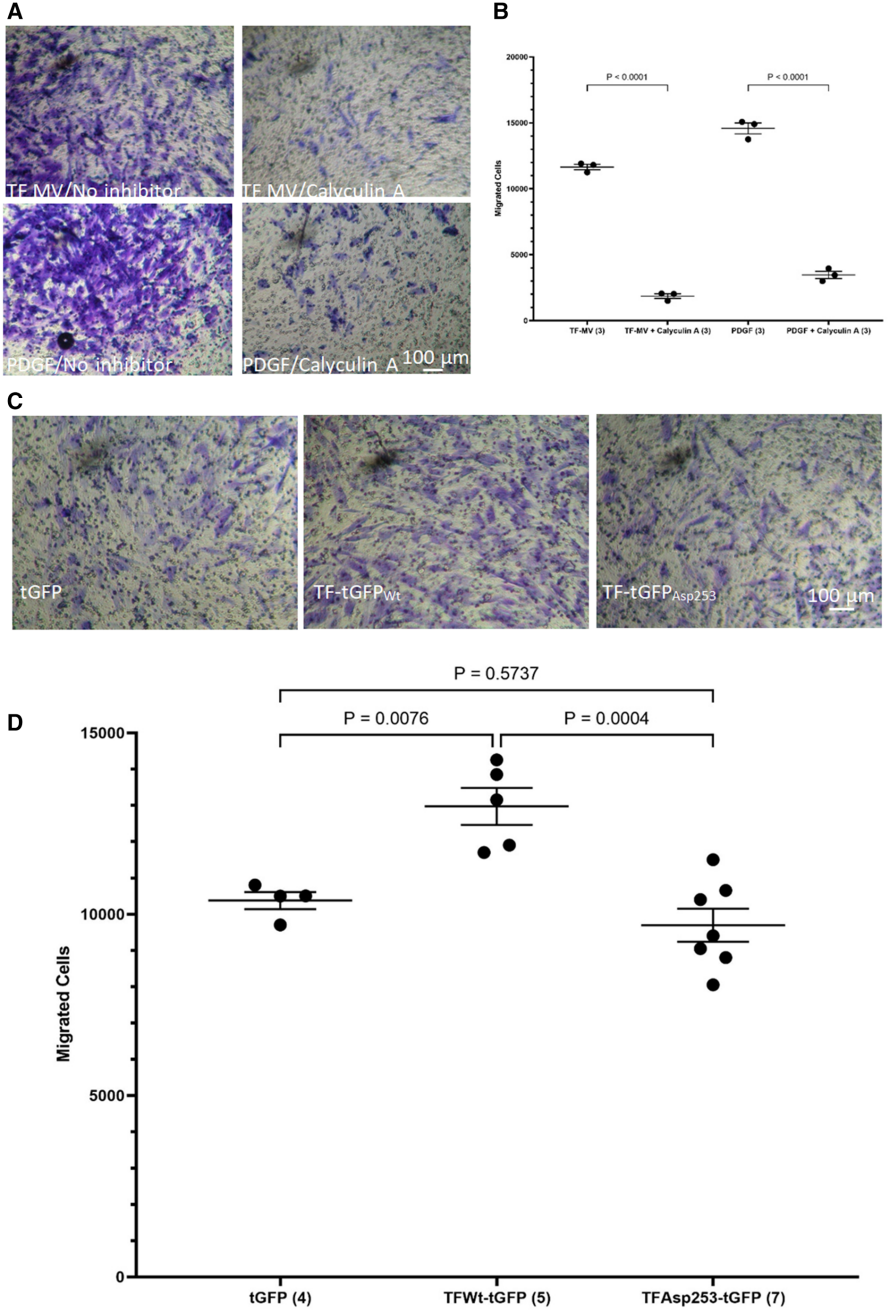
Examination of the influence of TF de-phosphorylation on HCASMC migration. HCASMC were treated with calyculin-A (1 nM) and cell migration stimulated with MV containing TF_Wt_-tGFP or PDGF-BB (10 ng/ml) at 37°C for 18 h. The cells on the upper side of the chamber were scraped off, stained with crystal violet and (**A**) photographed. (**B**) The numbers of migrating cells were determined by eluting the crystal violet and measuring the absorptions at 595 nm. Presented data include the calculated mean values from 3 experiments. HCASMC were transfected to express TF_Wt_-tGFP, TF_Asp253_-tGFP, or tGFP alone. Cell migration was induced by placing fVIIa (3 nM) in complete media (250 μl), in the lower chamber and incubated at 37°C for 18 h. The cells were then fixed, washed and the cells on the upper side of the chamber were then scraped off. (**C**) The cells were stained with crystal violet and photographed and (**D**) the numbers of migrating cells were determined by eluting the crystal violet and measuring the absorptions at 595 nm. Presented data include the calculated mean values ± the calculated standard error of the mean, from 4–7 experiments as shown at the base of each column.

While the incorporation of TF within the focal adhesion points may establish the direction of cell migration (44), focal adhesion disassembly is vital for trailing edge retraction. In the above experiments the stimulation of HCASMC with MV carrying TF_Asp253_-tGFP resulted in the retardation of cell migration. In addition, transfection of HCASMC with phospho-Ser253 peptide prevented the stimulation with MV carrying TF_Wt_-tGFP. To examine the involvement of TF-de-phosphorylation in chemotaxis, first PP2A phosphatase activity was inhibited in HCASMC (46). Pre-incubation of HCASMC with calyculin-A (1 nM) prior to stimulation with MV carrying TF_Wt_-tGFP severely suppressed TF-induced cell migration (Figures 7A,B). However, cell migration in response to PDGF-BB was also affected by inclusion of calyculin-A. Therefore, no definitive conclusions were derived from these experiments. To further explore the possibility that the TF-de-phosphorylation is required for migration, HCASMC were transfected to express TF_Wt_-tGFP, TF_Asp253_-tGFP, or tGFP alone and the rate of cell migration in response to fVIIa, examined. Since unlike TF_Wt_-tGFP, TF_Asp253_-tGFP retains the ability to interact with filamin A, the observed reduction in the rate of HCASMC migration suggests that TF-de-phosphorylation is likely to be essential as part of the chemoattractant or migratory mechanism (Figures 7C,D).

### 3.6 Induction of HCASMC migration requires focal adhesion kinase activity but is independent of receptor-kinase activity

**Figure 8:**
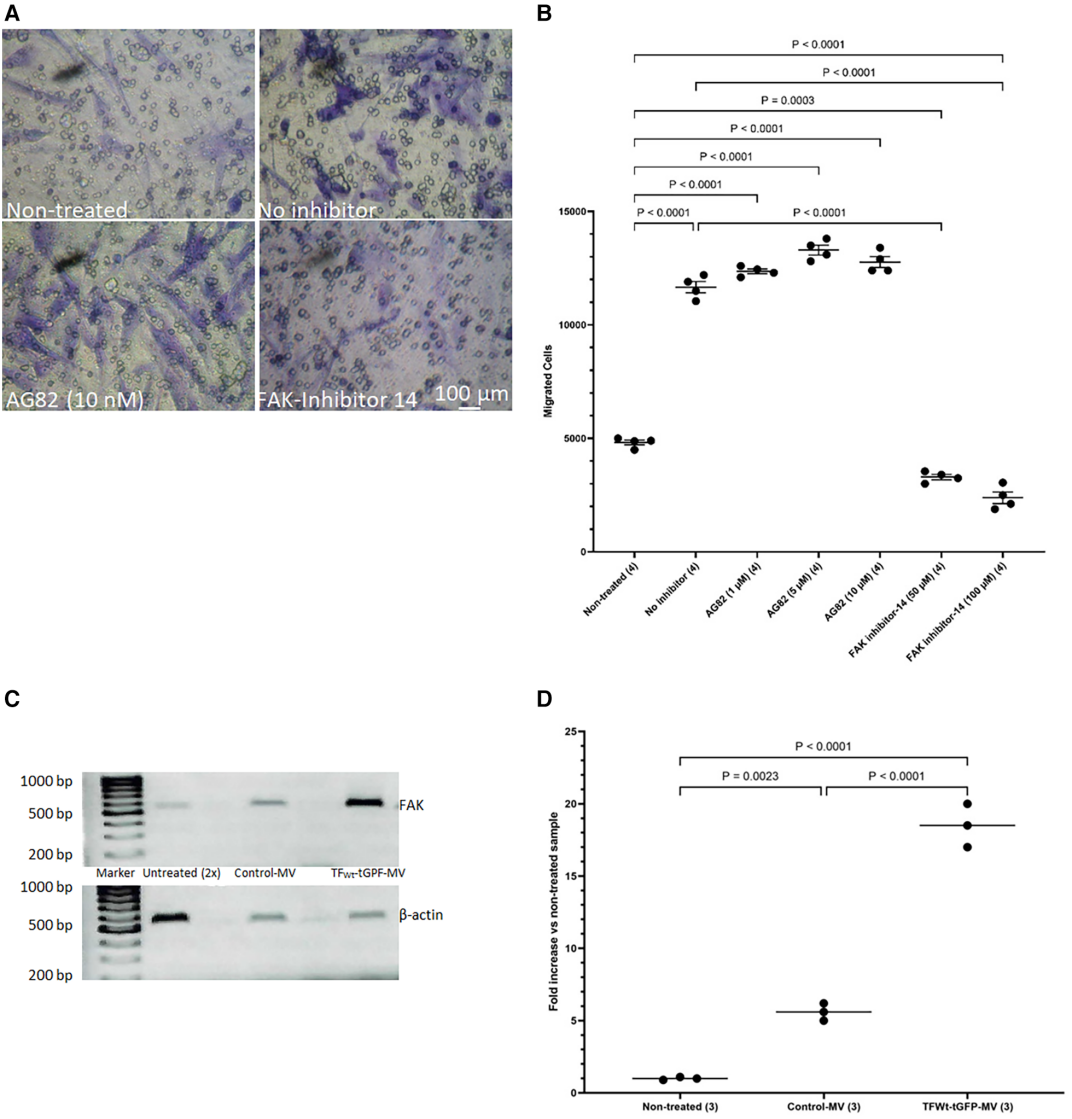
Examination of the influence of FAK inhibition on migration, and upregulation of *FAK* by TF. HCASMC were pre-incubated with either FAK inhibitor-14 (0–100 μM) to inhibit FAK autophosphorylation, or AG82 that prevents receptor-kinase mediated pTyr125-FAK phosphorylation. HCASMC (3 × 10^4^) were incubated at 37°C with MV prepared from HCAEC expressing TF_Wt_-tGFP placed in the bottom chamber. After 18 h the upper side of the chamber were then scraped off, (**A**) stained with crystal violet and photographed and (**B**) the numbers of migrating cells were determined by eluting the crystal violet and measuring the absorptions at 595 nm. Presented data include the calculated mean values ± the calculated standard error of the mean, from 4 experiments. (**C**) HCASMC were incubated overnight with MV prepared from HCAEC expressing TF_Wt_-tGFP or control MV. Total RNA was isolated from 2 × 10^5^ cells and amplified by PCR, using primers for *FAK* and β*-actin* RT-PCR products were visualized on a 2% (w/v) agarose gel, stained with SYBR Green I (Micrographs are representative of 3 separate experiments; Note that double the volume of non-treated sample was loaded since the amplified *FAK* RNA was not observable at lower amounts). (**D**) The ratio of the of *FAK*: β*-actin* was calculated for each sample. Presented data include the calculated mean values from 3 experiments.

The above data suggest that HCASMC migration in response to TF may be distinct to that induced by growth factor engagement. To further decipher the mechanisms involved in TF-mediated cell migration and to differentiate between the TF-induced and growth factor/cytokine-promoted cell migration, HCASMC were pre-incubated with either FAK inhibitor-14 (0–100 μM; 1,2,4,5-benzenetetraamine tetrahydrochloride) to inhibit FAK autophosphorylation, or AG82 that prevents receptor-kinase mediated p125FAK tyrosine phosphorylation. Preincubation of cells with FAK inhibitor-14 inhibited the TF-MV mediated SMC migration at both concentrations used. However, pre-incubation of the cells with AG82 (up to 10 μM) did not interfere with TF-fVIIa induced cell migration (Figures 8A,B) but reduced response to PDGF-BB (not shown). Furthermore, examination of the expression of *FAK* mRNA, in HCASMC following incubation with TF_Wt_-tGFP containing MV, indicated the upregulation of *FAK* expression at 18 h post-treatment (Figures 8C,D), but not on incubation with control MV.

## 4 Discussion

The importance of the communication between vascular endothelial and SMCs in the promotion and regulation of repair, following injury or trauma to the blood vessel has been established (11–15). This crosstalk has also been implicated in vascular remodeling through induction of synthetic SMC leading to increased proliferation and migration (6, 13, 14). Additionally, the contributions of cell-derived microvesicles (MV) to these processes has been highlighted recently and a subject of interest (1, 6, 8, 12, 15, 18). The endothelial layer is the site of early damage during disease and consequently, the arising inflammatory signals relay instructions to the underlying cells within the intima. TF-containing microvesicles are released early as a means of initiating the coagulation mechanism following injury (1) but also act as means of promoting cellular processes leading to repair. However, prolonged exposure to TF has been implicated in a number of conditions, including vascular diseases (22, 23). Different influences of TF-containing microvesicles derived from SMCs, on endothelial dysfunction have previously been reported (12, 15). As stated above, injury and inflammation stimulate vascular SMCs to switch into a de-differentiated synthetic phenotype. The increased rate of synthetic VSMC migration promotes vascular repair, but inappropriate migration/proliferation can also lead to vascular deformity. This study aimed to decipher some of the influences exerted by endothelial TF-MV on SMC mobility.

Our data highlights the potential of MV as carriers for TF, transferring this cargo to VSMC to promote TF-dependent cell migration. This mechanism involves PAR2 activation which appears to be mainly activated by fVIIa-TF complex, rather than by fXa, and agrees with previous reports (33, 34). Addition of fVIIa alone induced a lower rate of cell migration since HCASMC were shown to express low levels of TF (50, 52–54). Interestingly, in the absence of exogenous TF, direct activation of PAR2 was not sufficient to accelerate cell migration suggesting the low-level engagement of cellular TF. Inhibition of protease activity of TF-fVIIa complex, either by pre-incubation of MV with HTF-1 antibody, or an inhibitory anti-fVIIa antibody reduced the rate of HCASMC migration to that observed in non-treated cells. Similarly, blocking PAR2 activation/signaling by pre-incubation of HCASMC with SAM11 antibody reduced the rate of HCASMC migration. The interaction of fVIIa with TF appears to promote the co-localization with PAR2 (28) resulting in the activation of PAR2 (60) and in turn induces a diverse number of downstream signaling mechanisms (33, 34, 61, 62). The majority of the reports also implicate the activation of RhoA as an essential step in the promotion of cell migration (63–65). This ability of RhoA to promote cytoskeletal reorganization points to a chemotactic function for PAR2 signaling. However, feedback mechanisms from PAR2 activation are also known to induce the phosphorylation of the cytoplasmic domain of TF at serine 253, by protein kinase C (46, 66) which as stated above, also promote signaling mechanisms by interaction with β1-integrin and through binding to filamin-A. However, blocking of the exosite on TF, using the 10H10 antibody had no influence on cell migration. Also unsurprisingly, inhibition of β1-integrin using the AIIB2 antibody completely abolished HCASMC adhesion/migration and was attributed to the disruption of the adhesion complex and possibly independent of TF. Therefore, while it is likely that the interaction of TF with β1-integrin contributes to cell migration by disrupting cellular adhesion to the extracellular matrix (67), the direct outcomes of signaling arising from this complex formation could not be established and was not pursued.

In contrast, deletion of the cytoplasmic domain leaving a procoagulant active TF significantly reduced the rate of HCASMC migration although the levels were above those observed in the non-treated control cells. Moreover, treatment of cells with recombinant TF was only capable of inducing migration when in conjunction with exogenous fVIIa. Together, these data suggest that the cytoplasmic domain of TF may act as a guide, determining the direction of the cell migration towards the point of contact with MV. Therefore, in agreement with previous studies (19, 43) both the cytoplasmic domain of TF and PAR2 activation appear to be required for the efficient induction of HCASMC migration. In this model, the PAR2 signaling provides the components for chemokinesis while the interaction of TF with filamin-A permits directional chemotaxis.

As stated before, TF has been shown to co-localize with filamin A at the leading edge of lamellipodia in migrating SMC (19, 39). Furthermore, the interaction of TF with filamin A is enhanced following the phosphorylation of the cytoplasmic domain of TF (43, 44), and is specifically mediated through phosphorylation of serine 253 (45). To further explore the role of TF phosphorylation, peptides corresponding to the cytoplasmic domain of TF, which contained different phosphorylation patterns were used as competitors. The ability of TF-MV to induce HCASMC migration was completely abolished in cells transfected with peptides when phosphorylated at position of serine 253, and was irrespective of the state of serine 258. These data agree with previous studies identifying the phosphorylation of serine 253 as an essential step in recruitment by filamin A (43, 45), permitting the association of TF with filamin A at the leading edge of cells (19, 39). Furthermore, expression of mutant form of filamin A devoid of repeat-24 alone, was sufficient to disrupt the response of HCASMC to TF-MV, identifying this domain as responsible for TF-mediated HCASMC migration. Interestingly, deletion of repeat-24 did not influence the migratory response of HCASMC towards PDGF-BB. Moreover, inhibition of FAK phosphorylation through growth factor receptor kinase activity did not influence TF-induced HCASMC migration. Therefore, the interaction with repeat-24 of filamin A may be a specific mechanism, used by a few unconventional receptors, including TF. The proximity of the repeat-24 domains in filamin A dimers may also explain the previous observations of TF dimerization on cell surface (68).

Stimulation of HCASMC with MV containing aspartate 253-substituted TF (TF_Asp253_-tGFP) to mimic phospho-serine 253 resulted in the retardation of cell migration. This was also confirmed by the reduction in the rate of HCASMC migration, on expression of TF_Asp253_-tGFP, when compared to cells expressing the wild type TF. One possible explanation may be that while the phosphorylation of TF at the leading-edge of the cell promotes its recruitment within the focal adhesion points, de-phosphorylation of TF at the trailing-edge, may be essential for the disengagement and release. Increased RhoA activity is known to occur following PAR2 activation, and by TF-fVIIa complex (19, 63, 64). Combined RhoA and ROCK activity is associated with the tail retraction of migrating cells (69), while RhoA-signaling via formin mDia 1 leads to lamellipodia formation (70–72). It has therefore been suggested that too much RhoA activity at the leading edge can inhibit lamellipodia extensions (73, 74). Additionally, membrane blebbing has been associated with directional migration during development (69). It is possible that the interaction of TF with filamin A alters the membrane-to-cortex attachment, subsequently encouraging a bleb-initiated direction for the migrating cells (69, 73). In contrast, prolonged TF activity as observed with TF_Asp253_, would significantly increase the rate of MV blebbing, and reduce the directionality of cell migration (69, 75). A better understanding the function of TF in VSMC migration would require detailed investigations on the influence of MV release from the cell on its migration, the differential regulation of RhoA by PAR2, and the possible function of TF de-phosphorylation in the dismantling of the focal adhesion complexes.

The above observations, together with upregulation of *FAK* mRNA expression by TF-MV suggests the enhanced migration may include the transformation of the cell phenotype. We previously showed that the expression of TF_Asp253_ mutants in EC, improves cell viability (76) while expression of TF_Ala253_ to prevent phosphorylation, induced cell apoptosis. These processes involve the activation of Src but are independent of FAK phosphorylation (77). Other TF-induced phenotypic alterations have also been associated with FAK recruitment (78, 79). Therefore, alterations in one specific marker, expressed only in contractile HCASMC phenotype were explored. Smoothelin-A and -B are specific markers of SMCs and have been reported to differentiate between contractile or synthetic phenotypes (80, 81). Smoothelin-B is specifically expressed in the vascular SMCs, particularly associated with muscular arteries, is lower in elastic arteries, and is absent in the capillaries, pericytic venules and small veins (82). Importantly, Smoothelin-B is rapidly downregulated following vascular damage and/or compromise. The expression of Smoothelin-B is then restored upon successful completion of remodeling to healthy tissue. However, the levels of smoothelin-B have been shown to remain low during chronic conditions for example, the course of atherosclerosis, or the development of restenosis (83–87). In our study, incubation of HCASMC with MV containing TF-tGFP or TF_Asp253_-tGFP almost completely eliminated the expression of vascular-specific Smoothelin-B while the truncated TF_ΔCT_-tGFP was partially effective (Figure 3). These reductions were similar to those observed on incubation of HCASMC with PDGF-BB which is also known to suppress the expression of Smoothelin-B (88). The gene for Smoothelin can be differentialy expressed as two separate transcripts (A & B). These are also regulated through separate repressor regions but the exact mechanisms are unclear (89). Therefore, while our data suggests a change in the cell morphology, a comprehensive study is required to establish the influence of TF-mediated signaling on VSMC phenotype markers. Therefore, we have refrained from speculating about cell morphological changes which are beyond the remit of the study.

In conclusion, this study has shown that Ser253-phosphorylated TF and fVIIa released as MV-cargo by EC in response to inflammation or injury, can act as messengers for rapid promotion of VSMC migration. The underlying mechanism requires both the activation of PAR2 by TF-fVIIa, as well as the interaction of TF with filamin A, involving repeat-24 on the latter protein. These pathways are initiated following incorporation of TF into the cell membrane but subsequently diverge. This mechanism ensures a rapid and proportional VSMC response, gauged to the level of inflammation conveyed by the endothelium, and appropriate for restoring normal arterial integrity/function. However, prolonged inflammatory response by the endothelium could contribute to the development of vascular abnormalities associated with chronic diseases.

## Data Availability

The original contributions presented in the study are included in the article/**supplementary materials**, further inquiries can be directed to the corresponding author.

## References

[1] Badimon L Suades R Arderiu G Peña E Chiva-Blanch G Padró T. Microvesicles in atherosclerosis and angiogenesis: from bench to bedside and reverse. Front Cardiovasc Med. (2017) 4:77. 10.3389/fcvm.2017.00077 29326946 PMC5741657

[2] Mulvany MJ Baumbach GL Aalkjaer C Heagerty AM Korsgaard N Schiffrin EL Vascular remodeling. Hypertension. (1996) 28(3):505–6. 8794840

[3] Santos-Gallego CG Picatoste B Badimón JJ. Pathophysiology of acute coronary syndrome. Curr Atheroscler Rep. (2014) 16(4):401. 10.1007/s11883-014-0401-9 24504549

[4] Plekhanova OS Parfenova EV Tkachuk VA. Mechanisms of vascular remodeling following arterial injury. Kardiologiia. (2015) 55(7):63–77. 10.18565/cardio.2015.7.63-77 26688928

[5] Shi N Mei X Chen SY. Smooth muscle cells in vascular remodeling. Arterioscler Thromb Vasc Biol. (2019) 39(12):e247–52. 10.1161/ATVBAHA.119.312581 31770033 PMC6937202

[6] Méndez-Barbero N Gutiérrez-Muñoz C Blanco-Colio LM. Cellular crosstalk between endothelial and smooth muscle cells in vascular wall remodeling. Int J Mol Sci. (2021) 22(14):7284. 10.3390/ijms22147284 34298897 PMC8306829

[7] Krankel N Luscher TF Landmesser U. Novel insights into vascular repair mechanisms. Curr Pharm Des. (2014) 20(14):2430–8. 10.2174/13816128113199990478 23844814

[8] Morel O Morel N Jesel L Freyssinet JM Toti F. Microparticles: a critical component in the nexus between inflammation, immunity, and thrombosis. Semin Immunopathol. (2011) 33(5):469–86. 10.1007/s00281-010-0239-3 21866419

[9] Lin ZB Ci HB Li Y Cheng TP Liu DH Wang YS Endothelial microparticles are increased in congenital heart diseases and contribute to endothelial dysfunction. J Transl Med. (2017) 15(1):4. 10.1186/s12967-016-1087-2 28049487 PMC5210308

[10] Li M Qian M Kyler K Xu J. Endothelial-Vascular smooth muscle cells interactions in atherosclerosis. Front Cardiovasc Med. (2018) 5:151. 10.3389/fcvm.2018.00151 30406116 PMC6207093

[11] Gao Y Chen T Raj JU. Endothelial and smooth muscle cell interactions in the pathobiology of pulmonary hypertension. Am J Respir Cell Mol Biol. (2016) 54(4):451–60. 10.1165/rcmb.2015-0323TR 26744837 PMC4821060

[12] Stampfuss JJ Censarek P Fischer JW Schrör K Weber AA. Rapid release of active tissue factor from human arterial smooth muscle cells under flow conditions. Arterioscler Thromb Vasc Biol. (2006) 26(5):e34–37. 10.1161/atvb.26.5.1184 16528008

[13] Mause SF Ritzel E Deck A Vogt F Liehn EA. Endothelial progenitor cells modulate the phenotype of smooth muscle cells and increase their neointimal accumulation following vascular injury. Thromb Haemost. (2022) 122(3):456–69. 10.1055/s-0041-1731663 34214997

[14] Cao G Xuan X Hu J Zhang R Jin H Dong H. How vascular smooth muscle cell phenotype switching contributes to vascular disease. Cell Commun Signal. (2022) 20(1):180. 10.1186/s12964-022-00993-2 36411459 PMC9677683

[15] Jia LX Zhang WM Li TT Liu Y Piao CM Ma YC ER stress dependent microparticles derived from smooth muscle cells promote endothelial dysfunction during thoracic aortic aneurysm and dissection. Clin Sci (Lond). (2017) 131(12):1287–99. 10.1042/CS20170252 28468950 PMC5461939

[16] Hasenstab D Lea H Hart CE Lok S Clowes AW. Tissue factor overexpression in rat arterial neointima models thrombosis and progression of advanced atherosclerosis. Circulation. (2000) 101(22):2651–7. 10.1161/01.CIR.101.22.2651 10840019

[17] Ikeda U Hojo Y Shimada K. Tissue factor overexpression in rat arterial neointima models: thrombosis and progression of advanced atherosclerosis. Circulation. (2001) 103(10):E59. 10.1161/01.CIR.103.10.e59 11245662

[18] Ghrib F Brisset AC Dupouy D Terrisse AD Navarro C Cadroy Y The expression of tissue factor and tissue factor pathway inhibitor in aortic smooth muscle cells is up-regulated in synthetic compared to contractile phenotype. Thromb Haemost. (2002) 87(6):1051–6. 10.1055/s-0037-1613131 12083485

[19] Peña E Arderiu G Badimon L. Subcellular localization of tissue factor and human coronary artery smooth muscle cell migration. J Thromb Haemost. (2012) 10(11):2373–82. 10.1111/j.1538-7836.2012.04910.x 22938499

[20] Moons AH Levi M Peters RJ. Tissue factor and coronary artery disease. Cardiovasc Res. (2002) 53(2):313–25. 10.1016/S0008-6363(01)00452-7 11827681

[21] Lim XC Yatim SMJM Chong SY Wang X Tan SH Yang X Plasma tissue factor coagulation activity in post-acute myocardial infarction patients. Front Endocrinol (Lausanne). (2022) 13:1008329. 10.3389/fendo.2022.1008329 36213278 PMC9540383

[22] Grover SP Mackman N. Tissue factor in atherosclerosis and atherothrombosis. Atherosclerosis. (2020) 307:80–6. 10.1016/j.atherosclerosis.2020.06.003 32674807

[23] Tatsumi K Mackman N. Tissue factor and atherothrombosis. J Atheroscler Thromb. (2015) 22(6):543–9. 10.5551/jat.30940 26016513

[24] Hisada Y Mackman N. Tissue factor and cancer: regulation, tumor growth, and metastasis. Semin Thromb Hemost. (2019) 45(4):385–95. 10.1055/s-0039-1687894 31096306 PMC6546519

[25] Zelaya H Rothmeier AS Ruf W. Tissue factor at the crossroad of coagulation and cell signaling. J Thromb Haemost. (2018) 16(10):1941–52. 10.1111/jth.14246 30030891

[26] Collier ME Mah PM Xiao Y Maraveyas A Ettelaie C. Microparticle-associated tissue factor is recycled by endothelial cells resulting in enhanced surface tissue factor activity. Thromb Haemost. (2013) 110(5):966–76. 10.1160/TH13-01-0055 23945646

[27] Hansen CB Pyke C Petersen LC Rao LV. Tissue factor-mediated endocytosis, recycling, and degradation of factor VIIa by a clathrin-independent mechanism not requiring the cytoplasmic domain of tissue factor. Blood. (2001) 97(6):1712–20. 10.1182/blood.V97.6.1712 11238112

[28] Madkhali Y Rondon AMR Featherby S Maraveyas A Greenman J Ettelaie C. Factor VIIa regulates the level of cell-surface tissue factor through separate but cooperative mechanisms. Cancers (Basel. (2021) 13(15):3718. 10.3390/cancers13153718 34359618 PMC8345218

[29] Thiruvikraman SV Guha A Roboz J Taubman MB Nemerson Y Fallon JT. In situ localization of tissue factor in human atherosclerotic plaques by binding of digoxigenin-labeled factors VIIa and X. Lab Invest. (1996) 75(4):451–61. 8874378

[30] Gertz SD Fallon JT Gallo R Taubman MB Banai S Barry WL Hirudin reduces tissue factor expression in neointima after balloon injury in rabbit femoral and porcine coronary arteries. Circulation. (1998) 98(6):580–7. 10.1161/01.CIR.98.6.580 9714116

[31] Hatakeyama K Asada Y Marutsuka K Sato Y Kamikubo Y Sumiyoshi A. Localization and activity of tissue factor in human aortic atherosclerotic lesions. Atherosclerosis. (1997) 133(2):213–9. 10.1016/S0021-9150(97)00132-9 9298681

[32] Schecter AD Spirn B Rossikhina M Giesen PL Bogdanov V Fallon JT Release of active tissue factor by human arterial smooth muscle cells. Circ Res. (2000) 87(2):126–32. 10.1161/01.RES.87.2.126 10903996

[33] Marutsuka K Hatakeyama K Sato Y Yamashita A Sumiyoshi A Asada Y. Protease-activated receptor 2 (PAR2) mediates vascular smooth muscle cell migration induced by tissue factor/factor VIIa complex. Thromb Res. (2002) 107(5):271–6. 10.1016/S0049-3848(02)00345-6 12479889

[34] Siegbahn A Johnell M Nordin A Aberg M Velling T. TF/FVIIa transactivate PDGFRbeta to regulate PDGF-BB-induced chemotaxis in different cell types:involvement of Src and PLC. Arterioscler Thromb Vasc Biol. (2008) 28(1):135–41. 10.1161/ATVBAHA.107.155754 17991872

[35] Hergenreider E Heydt S Tréguer K Boettger T Horrevoets AJ Zeiher AM Atheroprotective communication between endothelial cells and smooth muscle cells through miRNAs. Nat Cell Biol. (2012) 14(3):249–56. 10.1038/ncb2441 22327366

[36] Calderwood DA Huttenlocher A Kiosses WB Rose DM Woodside DG Schwartz MA Increased filamin binding to beta-integrin cytoplasmic domains inhibits cell migration. Nat Cell Biol. (2001) 3:1060–8. 10.1038/ncb1201-1060 11781567

[37] Woo MS Ohta Y Rabinovitz I Stossel TP Blenis J. Ribosomal S6 kinase (RSK) regulates phosphorylation of filamin A on an important regulatory site. Mol Cell Biol. (2004) 24:3025–35. 10.1128/MCB.24.7.3025-3035.2004 15024089 PMC371131

[38] Savoy RM Ghosh PM. The dual role of filamin A in cancer: can't live with (too much of) it, can't live without it. Endocr Relat Cancer. (2013) 20(6):R341–56. 10.1530/ERC-13-0364 24108109 PMC4376317

[39] Müller M Albrecht S Gölfert F Hofer A Funk RH Magdolen V Localization of tissue factor in actin-filament-rich membrane areas of epithelial cells. Exp Cell Res. (1999) 248(1):136–47. 10.1006/excr.1999.4395 10094821

[40] Taubman MB Marmur JD Rosenfield CL Guha A Nichtberger S Nemerson Y. Agonist-mediated tissue factor expression in cultured vascular smooth muscle cells. Role of Ca2+mobilization and protein kinase C activation. J Clin Invest. (1993) 91:547–52. 10.1172/JCI116234 8432863 PMC287977

[41] Jiang X Bailly MA Panetti TS Cappello M Konigsberg WH Bromberg ME. Formation of tissue factor-factor VIIa-factor Xa complex promotes cellular signaling and migration of human breast cancer cells. J Thromb Haemost. (2004) 2:93–101. 10.1111/j.1538-7836.2004.00545.x 14717972

[42] Bandaru S Ala C Zhou AX Akyürek LM. Filamin A regulates cardiovascular remodeling. Int J Mol Sci. (2021) 22(12):6555. 10.3390/ijms22126555 34207234 PMC8235345

[43] Ott I Fischer EG Miyagi Y Mueller BM Ruf W. A role for tissue factor in cell adhesion and migration mediated by interaction with actin-binding protein 280. J Cell Biol. (1998) 140:1241–53. 10.1083/jcb.140.5.1241 9490735 PMC2132689

[44] Ott I Michaelis C Schuermann M Steppich B Seitz I Dewerchin M Vascular remodeling in mice lacking the cytoplasmic domain of tissue factor. Circ Res. (2005) 97:293–8. 10.1161/01.RES.0000177533.48483.12 16020755

[45] Collier MEW Ettelaie C Goult BT Maraveyas A Goodall AH. Investigation of the filamin A-dependent mechanisms of tissue factor incorporation into microvesicles. Thromb Haemost. (2017) 117(11):2034–44. 10.1160/TH17-01-0009 29044292

[46] Collier ME Ettelaie C. Regulation of the incorporation of tissue factor into microparticles by serine phosphorylation of the cytoplasmic domain of tissue factor. J Biol Chem. (2011) 286(14):11977–84. 10.1074/jbc.M110.195214 21310953 PMC3069400

[47] Collier ME Ettelaie C. Induction of endothelial cell proliferation by recombinant and microparticle-tissue factor involves beta1-integrin and extracellular signal regulated kinase activation. Arterioscler Thromb Vasc Biol. (2010) 30(9):1810–7. 10.1161/ATVBAHA.110.211854 20616308

[48] Ettelaie C Collier ME Maraveyas A Ettelaie R. Characterization of physical properties of tissue factor-containing microvesicles and a comparison of ultracentrifuge-based recovery procedures. J Extracell Vesicles. (2014) 3:23592. 10.3402/jev.v3.23592 PMC4134674 25206957

[49] Collier ME Maraveyas A Ettelaie C. Filamin-A is required for the incorporation of tissue factor into cell-derived microvesicles. Thromb Haemost. (2014) 111(4):647–55. 10.1160/TH13-09-0769 24258684

[50] Li C Collier ME Frentzou GA Greenman J Ettelaie C. Investigation of the mechanisms of tissue factor-mediated evasion of tumour cells from cellular cytotoxicity. Cancer Immunol Immunother. (2008) 57(9):1347–55. 10.1007/s00262-008-0469-6 18297283 PMC11029821

[51] Pradier A Ettelaie C. The influence of exogenous tissue factor on the regulators of proliferation and apoptosis in endothelial cells. J Vasc Res. (2008) 45(1):19–32. 10.1159/000109074 17898544

[52] Kraler S Libby P Evans PC Akhmedov A Schmiady MO Reinehr M The internal mammary artery and its resilience to atherogenesis: shifting from risk to resistance to address unmet needs. Arterioscler Thromb Vasc Biol. (2021) 41(8):2237–51. 10.1161/ATVBAHA.121.316256 34107731 PMC8299999

[53] Li J Chen T Wang DM Song YF Hong M. Annexin A5 inhibits homocysteine-induced tissue factor expression and activity in vascular smooth muscle cells. Zhonghua Xin Xue Guan Bing Za Zhi. (2009) 37(11):1039–43. 20137335

[54] Schecter AD Giesen PL Taby O Rosenfield CL Rossikhina M Fyfe BS Tissue factor expression in human arterial smooth muscle cells. TF is present in three cellular pools after growth factor stimulation. J Clin Invest. (1997) 100(9):2276–85. 10.1172/JCI119765 9410905 PMC508423

[55] Rodgers GM Greenberg CS Shuman MA. Characterization of the effects of cultured vascular cells on the activation of blood coagulation. Blood. (1983) 61(6):1155–62. 10.1182/blood.V61.6.1155.1155 6551179

[56] Featherby S Madkhali Y Maraveyas A Ettelaie C. Apixaban suppresses the release of TF-positive microvesicles and restrains cancer cell proliferation through directly inhibiting TF-fVIIa activity. Thromb Haemost. (2019) 119(9):1419–32. 10.1055/s-0039-1692682 31266079

[57] Madkhali Y Featherby S Collier ME Maraveyas A Greenman J Ettelaie C. The ratio of factor VIIa:tissue factor content within microvesicles determines the differential influence on endothelial cells. TH Open. (2019) 3(2):e132–45. 10.1055/s-0039-1688934 31259295 PMC6598090

[58] Carson SD Ross SE Bach R Guha A. An inhibitory monoclonal antibody against human tissue factor. Blood. (1987) 70(2):490–3. 10.1182/blood.V70.2.490.490 3607285

[59] Versteeg HH Schaffner F Kerver M Petersen HH Ahamed J Felding-Habermann B Inhibition of tissue factor signaling suppresses tumor growth. Blood. (2008) 111(1):190–9. 10.1182/blood-2007-07-101048 17901245 PMC2200804

[60] Camerer E Huang W Coughlin SR. Tissue factor- and factor X-dependent activation of protease-activated receptor 2 by factor VIIa. Proc Natl Acad Sci USA. (2000) 97(10):5255–60. 10.1073/pnas.97.10.5255 10805786 PMC25815

[61] Böhm A Flößer A Ermler S Fender AC Lüth A Kleuser B Factor-Xa-induced mitogenesis and migration require sphingosine kinase activity and S1P formation in human vascular smooth muscle cells. Cardiovasc Res. (2013) 99(3):505–13. 10.1093/cvr/cvt112 23658376

[62] Zhu T Mancini JA Sapieha P Yang C Joyal JS Honoré JC Cortactin activation by FVIIa/tissue factor and PAR2 promotes endothelial cell migration. Am J Physiol Regul Integr Comp Physiol. (2011) 300(3):R577–585. 10.1152/ajpregu.00137.2010 21160057

[63] Vouret-Craviari V Grall D Van Obberghen-Schilling E. Modulation of Rho GTPase activity in endothelial cells by selective proteinase-activated receptor (PAR) agonists. J Thromb Haemost. (2003) 1(5):1103–11. 10.1046/j.1538-7836.2003.00238.x 12871383

[64] Greenberg DL Mize GJ Takayama TK. Protease-activated receptor mediated RhoA signaling and cytoskeletal reorganization in LNCaP cells. Biochemistry. (2003) 42(3):702–9. 10.1021/bi027100x 12534282

[65] Sriwai W Mahavadi S Al-Shboul O Grider JR Murthy KS. Distinctive G protein-dependent signaling by protease-activated receptor 2 (PAR2) in smooth muscle: feedback inhibition of RhoA by cAMP-independent PKA. PLoS One. (2013) 8(6):e66743. 10.1371/journal.pone.0066743 23825105 PMC3688948

[66] Ahamed J Ruf W. Protease-activated receptor 2-dependent phosphorylation of the tissue factor cytoplasmic domain. J Biol Chem. (2004) 279(22):23038–44. 10.1074/jbc.M401376200 15039423

[67] Dorfleutner A Hintermann E Tarui T Takada Y Ruf W. Cross-talk of integrin alpha3beta1 and tissue factor in cell migration. Mol Biol Cell. (2004) 15(10):4416–25. 10.1091/mbc.e03-09-0640 15254262 PMC519137

[68] Doñate F Kelly CR Ruf W Edgington TS. Dimerization of tissue factor supports solution-phase autoactivation of factor VII without influencing proteolytic activation of factor X. Biochemistry. (2000) 39(37):11467–76. 10.1021/bi000986p 10985793

[69] Charras G Paluch E. Blebs lead the way: how to migrate without lamellipodia. Nat Rev Mol Cell Biol. (2008) 9(9):730–6. 10.1038/nrm2453 18628785

[70] Ridley AJ Schwartz MA Burridge K Firtel RA Ginsberg MH Borisy G Cell migration: integrating signals from front to back. Science. (2003) 302(5651):1704–9. 10.1126/science.1092053 14657486

[71] Heasman SJ Ridley AJ. Multiple roles for RhoA during T cell transendothelial migration. Small GTPases. (2010) 1(3):174–9. 10.4161/sgtp.1.3.14724 21686273 PMC3116607

[72] Heasman SJ Carlin LM Cox S Ng T Ridley AJ. Coordinated RhoA signaling at the leading edge and uropod is required for T cell transendothelial migration. J Cell Biol. (2010) 190(4):553–63. 10.1083/jcb.201002067 20733052 PMC2928012

[73] Ridley AJ. Life at the leading edge. Cell. (2011) 145(7):1012–22. 10.1016/j.cell.2011.06.010 21703446

[74] Petrie RJ Yamada KM. At the leading edge of three-dimensional cell migration. J Cell Sci. (2012) 125(Pt 24):5917–26. 10.1242/jcs.093732 23378019 PMC4067260

[75] Diz-Muñoz A Krieg M Bergert M Ibarlucea-Benitez I Muller DJ Paluch E Control of directed cell migration in vivo by membrane-to-cortex attachment. PLoS Biol. (2010) 8(11):e1000544. 10.1371/journal.pbio.1000544 21151339 PMC2994655

[76] ElKeeb AM Collier ME Maraveyas A Ettelaie C. Accumulation of tissue factor in endothelial cells induces cell apoptosis, mediated through p38 and p53 activation. Thromb Haemost. (2015) 114(2):364–78. 10.1160/TH14-09-0795 25903973

[77] Ethaeb AM Mohammad MA Madkhali Y Featherby S Maraveyas A Greenman J Accumulation of tissue factor in endothelial cells promotes cellular apoptosis through over-activation of Src1 and involves β1-integrin signaling. Apoptosis. (2020) 25(1-2):29–41. 10.1007/s10495-019-01576-2 31654241 PMC6965344

[78] Ünlü B Kocatürk B Rondon AMR Lewis CS Swier N van den Akker RFP Integrin regulation by tissue factor promotes cancer stemness and metastatic dissemination in breast cancer. Oncogene. (2022) 41(48):5176–85. 10.1038/s41388-022-02511-7 36271029

[79] Sovershaev TA Unruh D Sveinbjørnsson B Fallon JT Hansen JB Bogdanov VY A novel role of bone morphogenetic protein-7 in the regulation of adhesion and migration of human monocytic cells. Thromb Res. (2016) 147:24–31. 10.1016/j.thromres.2016.09.018 27669124

[80] van Eys GJ Niessen PM Rensen SS. Smoothelin in vascular smooth muscle cells. Trends Cardiovasc Med. (2007) 17(1):26–30. 10.1016/j.tcm.2006.11.001 17210475

[81] Wehrens XH Mies B Gimona M Ramaekers FC Van Eys GJ Small JV. Localization of smoothelin in avian smooth muscle and identification of a vascular-specific isoform. FEBS Lett. (1997) 405(3):315–20. 10.1016/S0014-5793(97)00207-X 9108311

[82] van der Loop FT Gabbiani G Kohnen G Ramaekers FC van Eys GJ. Differentiation of smooth muscle cells in human blood vessels as defined by smoothelin, a novel marker for the contractile phenotype. Arterioscler Thromb Vasc Biol. (1997) 17(4):665–71. 10.1161/01.ATV.17.4.665 9108778

[83] Christen T Verin V Bochaton-Piallat M Popowski Y Ramaekers F Debruyne P Mechanisms of neointima formation and remodeling in the porcine coronary artery. Circulation. (2001) 103(6):882–8. 10.1161/01.CIR.103.6.882 11171799

[84] Bär H Wende P Watson L Denger S van Eys G Kreuzer J Smoothelin is an indicator of reversible phenotype modulation of smooth muscle cells in balloon-injured rat carotid arteries. Basic Res Cardiol. (2002) 97(1):9–16. 10.1007/s395-002-8382-z 11998981

[85] Maeng M Mertz H Nielsen S van Eys GJ Rasmussen K Espersen GT. Adventitial myofibroblasts play no major role in neointima formation after angioplasty. Scand Cardiovasc J. (2003) 37(1):34–42. 10.1080/14017430310007018 12745801

[86] Johnson JL van Eys GJ Angelini GD George SJ. Injury induces dedifferentiation of smooth muscle cells and increased matrix-degrading metalloproteinase activity in human saphenous vein. Arterioscler Thromb Vasc Biol. (2001) 21(7):1146–51. 10.1161/hq0701.092106 11451743

[87] Hao H Gabbiani G Camenzind E Bacchetta M Virmani R Bochaton-Piallat ML. Phenotypic modulation of intima and media smooth muscle cells in fatal cases of coronary artery lesion. Arterioscler Thromb Vasc Biol. (2006) 26(2):326–32. 10.1161/01.ATV.0000199393.74656.4c 16339500

[88] Tharp DL Wamhoff BR Turk JR Bowles DK. Upregulation of intermediate- conductance Ca2+-activated K+ channel (IKCa1) mediates phenotypic modulation of coronary smooth muscle. Am J Physiol Heart Circ Physiol. (2006) 291(5):H2493–2503. 10.1152/ajpheart.01254.2005 16798818

[89] Rensen SS Thijssen VL De Vries CJ Doevendans PA Detera-Wadleigh SD Van Eys GJ. Expression of the smoothelin gene is mediated by alternative promoters. Cardiovasc Res. (2002) 55(4):850–63. 10.1016/S0008-6363(02)00491-1 12176134

