# Supplementary material for: Endothelial-derived microvesicles promote pro-migratory cross-talk with smooth muscle cells by a mechanism requiring tissue factor and PAR2 activation
Source: Front Cardiovasc Med. 2024 Jun 20;11:1365008. doi: 10.3389/fcvm.2024.1365008 (PMC11222581; doi:10.3389/fcvm.2024.1365008)
Supplement: Supplementary file 1 [file Datasheet1.docx]

Non-treated HTF-1 treated

Supplemental Figure 1- Human coronary artery endothelial cells (HCAEC) were transfected with 0.5 µg of pCMV6-Ac-TF-tGFP plasmid variants using TransIT-2020 according to the manufacturer’s instructions and permitted to express the proteins for 48 h prior to use. Cells were adapted to serum-free medium (SFM) for 1 h and the release of TF-containing microvesicles was induced by incubation with PAR2 agonist peptide (PAR2-AP); SLIGKV (20 µM). The conditioned media were cleared of any cell debris by centrifuging for 10 min at 2,500*g* on a microcentrifuge. The samples (1 ml aliquots) were then placed in 11×34 mm polycarbonate centrifuge tubes (Beckman Coulter), and the cell-derived microvesicles were sedimented at 100,000*g* on a TL-100 ultracentrifuge at 20°C, using a TLA 100.2 rotor (Beckman) for 1 h. Sedimented microvesicles were washed with PBS and re-centrifuged resuspended in pre-filtered (0.1 µm) PBS (200 µl). Samples of MV were pre-incubated with a mouse anti-human-TF antibody, HTF-1 (20 µg/ml) to block TF-fVIIa protease/procoagulant activity. The numbers and size distributions of the isolated microvesicles were examined by nanoparticle tracking analysis (NTA) using Nano-Sight LM10 and NTA software (NanoSight Ltd, Amesbury, UK). The Nanosight instrument was calibrated using FluoSpheres® carboxylate-modified microspheres with diameters of 0.1 μm and 1.0 μm (Invitrogen). Microvesicle samples were diluted 1:5 in 0.1 μm-filtered PBS and analysed using the NanoSight LM10 by tracking particles over 60 seconds using a camera level of 12 and shutter speed of 21.26 frames/s.


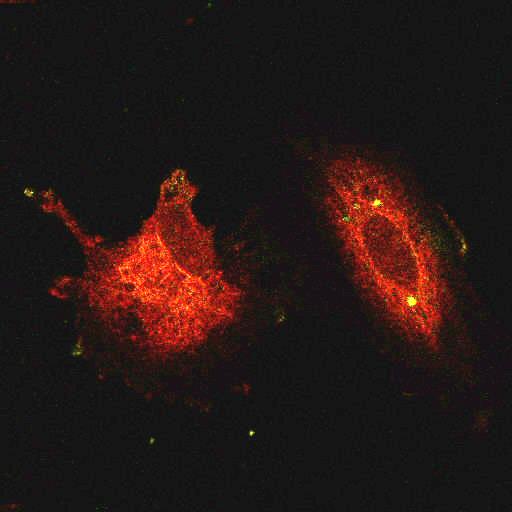

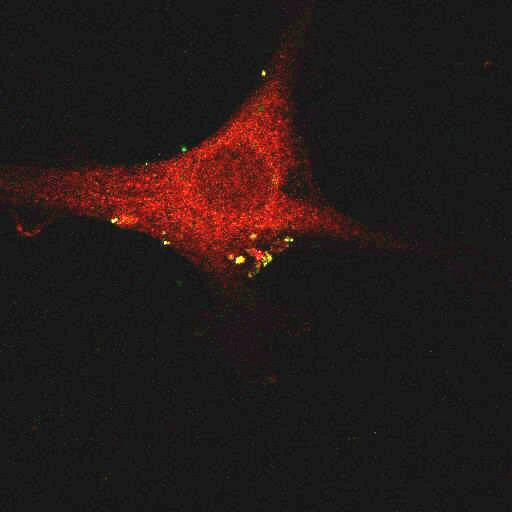

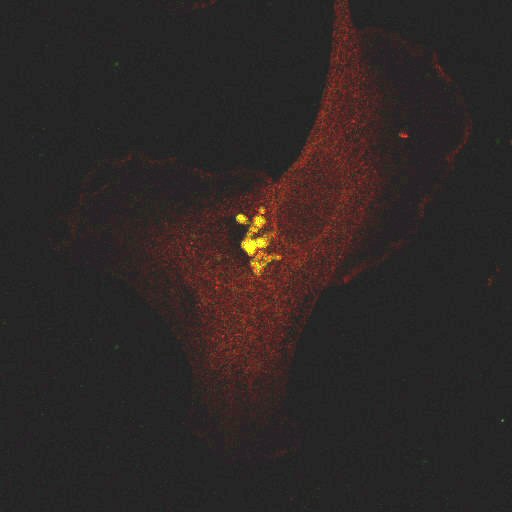

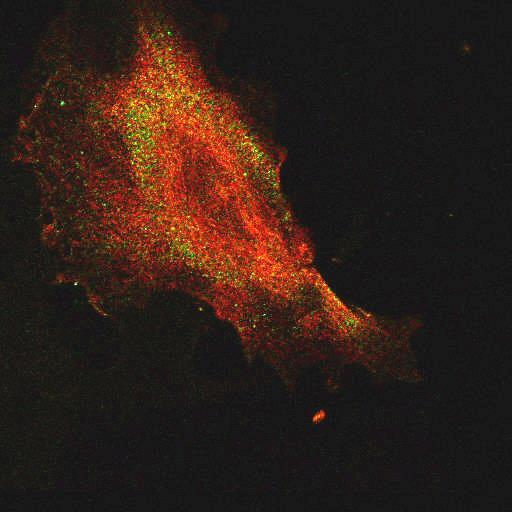


0 min 15 min

30 min 60 min

Supplemental Figure 2- HCASMC (3 x 10^4^) were seeded out into glass based dishes (μ-Dish 35 mm) and incubated with MV derived from the transfected HCAEC expressing TF_Wt_-tGFP, for up to 60 min. The cells were then washed three times with PBS and fixed with 4% (v/v) paraformaldehyde for 15 min. The cells were washed again and permeabilised with Triton X-100 0.1% (v/v) in PBS, for 5 min. The cells were labelled with rabbit anti-filamin A antibody (EP2405Y, Epitomics Inc, Burlingame, CA, USA) diluted 1:3000 v:v and probed with an anti-rabbit NorthernLights 637-conjugated antibody (R&D Systems, Abingdon, UK). The samples were analysed by confocal microscopy at room temperature using a Zeiss LSM 710 confocal microscope with a ×63 water immersion objective and images were acquired using the ZEN software (Carl Zeiss Ltd, Welwyn Garden City, UK).


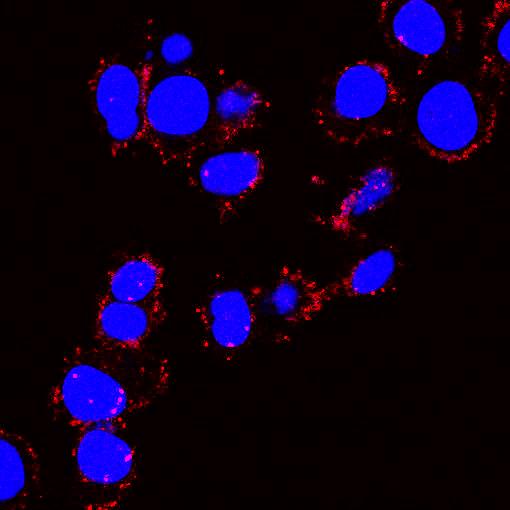

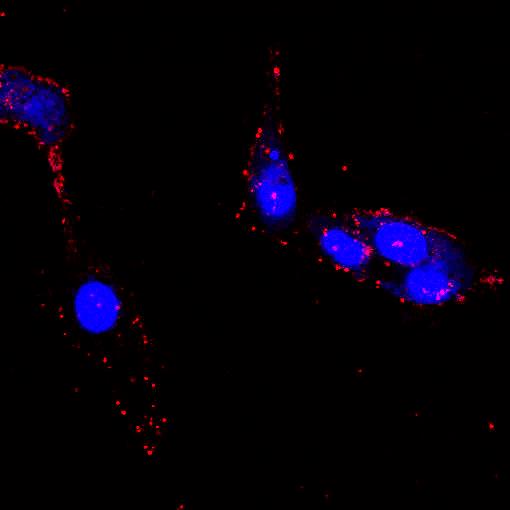


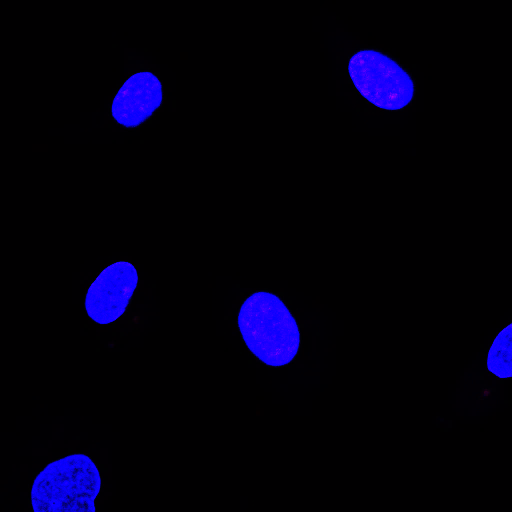


Anti-TF antibody (10H10) + Anti-Filamin A Anti-tGFP antibody (2HB) + Anti-Filamin A

Isotype IgG + Anti-Filamin A

Supplemental Figure 3- HCASMC (3 x 10^4^) were seeded out into glass based dishes (μ-Dish 35 mm) and incubated with MV derived from the transfected HCAEC expressing TF_Wt_-tGFP, for 30 min. The cells were then washed three times with PBS and fixed with 4% (v/v) paraformaldehyde for 15 min. The cells were washed again and permeabilised with Triton X-100 0.1% (v/v) in PBS, for 5 min. All samples were blocked with Duolink blocking buffer for 1 h and incubated overnight with combinations of antibodies as follows, at 4°C. The proximity between c-terminal of filamin A and TF-tGFP was examined using a rabbit anti-filamin A antibody against the c-terminal (EP2405Y; 2 μg/ml) together with either a mouse anti-TF antibody (10H10; 5 μg/ml), a mouse anti-tGFP antibody (2HB; 5 μg/ml; OriGene, Rockville, USA) or an isotype IgG (5 μg/ml). The antibodies were diluted in the provided antibody diluent buffer. The cells were washed three times with PBS and PLA performed according to the manufacturer’s instructions (Sigma Chemical Company, Poole, UK). The cells were labelled with DAPI (2 μg/ml). Images were acquired using a Zeiss LSM 710 confocal microscope with a ×63 water immersion objective and images were acquired using the ZEN software (Carl Zeiss Ltd, Welwyn Garden City, UK). (The micrographs are representative of 5 fields of view from 3 experiments, RED= PLA incidences; BLUE = DAPI).


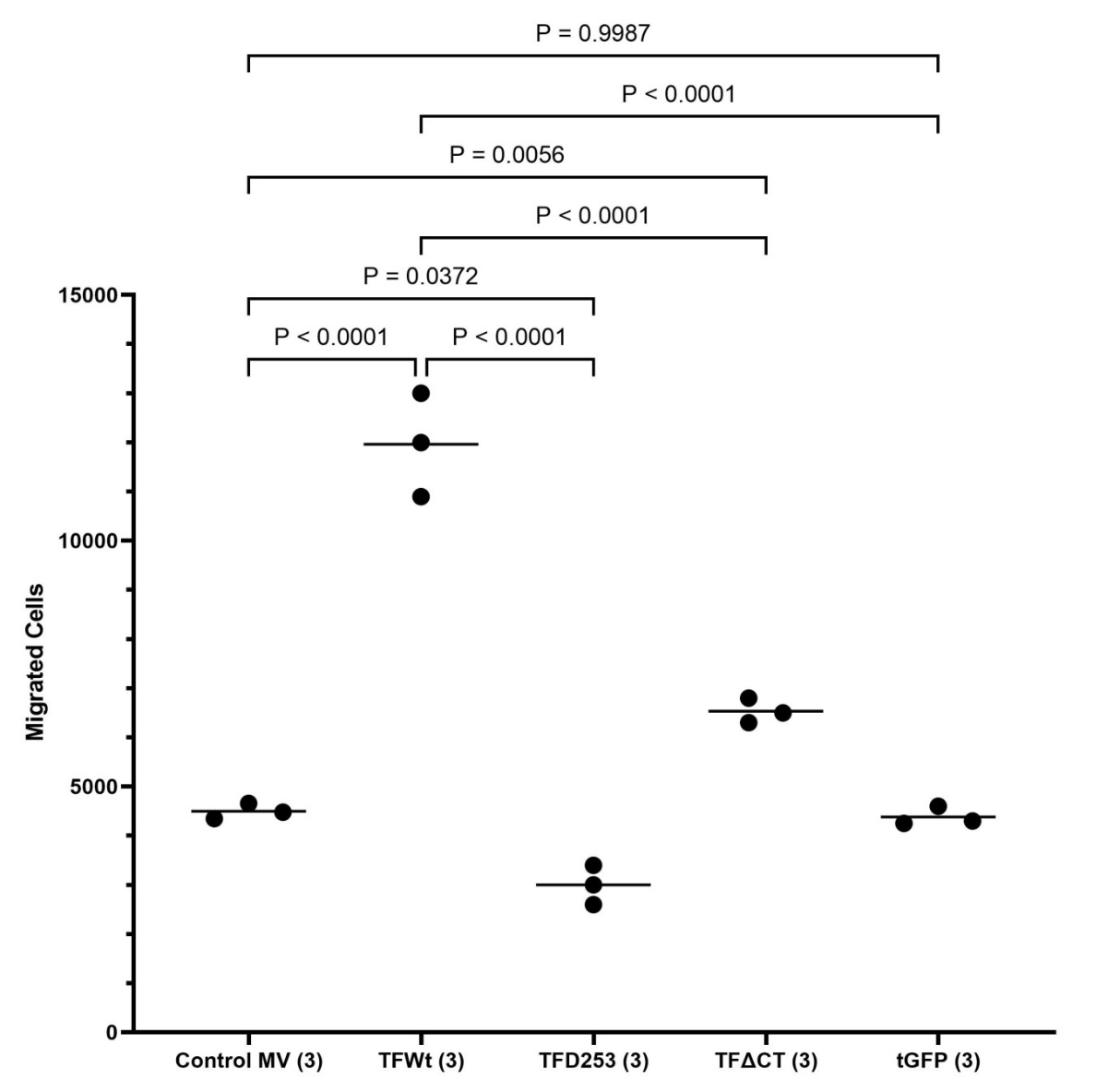


Supplemental Figure 4- MV were prepared from ECV304 (5 × 10^5^) transfected to express TF_Wt_-tGFP, TF_Asp253_-tGFP, TF_ΔCT_-tGFP or tGFP alone. HCASMC (3 × 10^4^ in 250 μl of media) were placed in the upper chamber of Boyden chambers. Complete media (250 μl), containing the isolated MV, was placed in the lower chamber and incubated at 37˚C for 18 h. After incubation, the cells were fixed, washed with PBS and the cells on the upper side of the chamber were then scraped off. The cells were stained with crystal violet and the numbers of migrating cells were determined by eluting the crystal violet and measuring the absorptions at 595 nm. Presented data include the calculated mean values from 3 biological repeats. Statistical analysis was carried out using the GraphPad Prism version 9.0 and significance was determined using one-way ANOVA (analysis of variance) and Tukey’s honesty significance test.


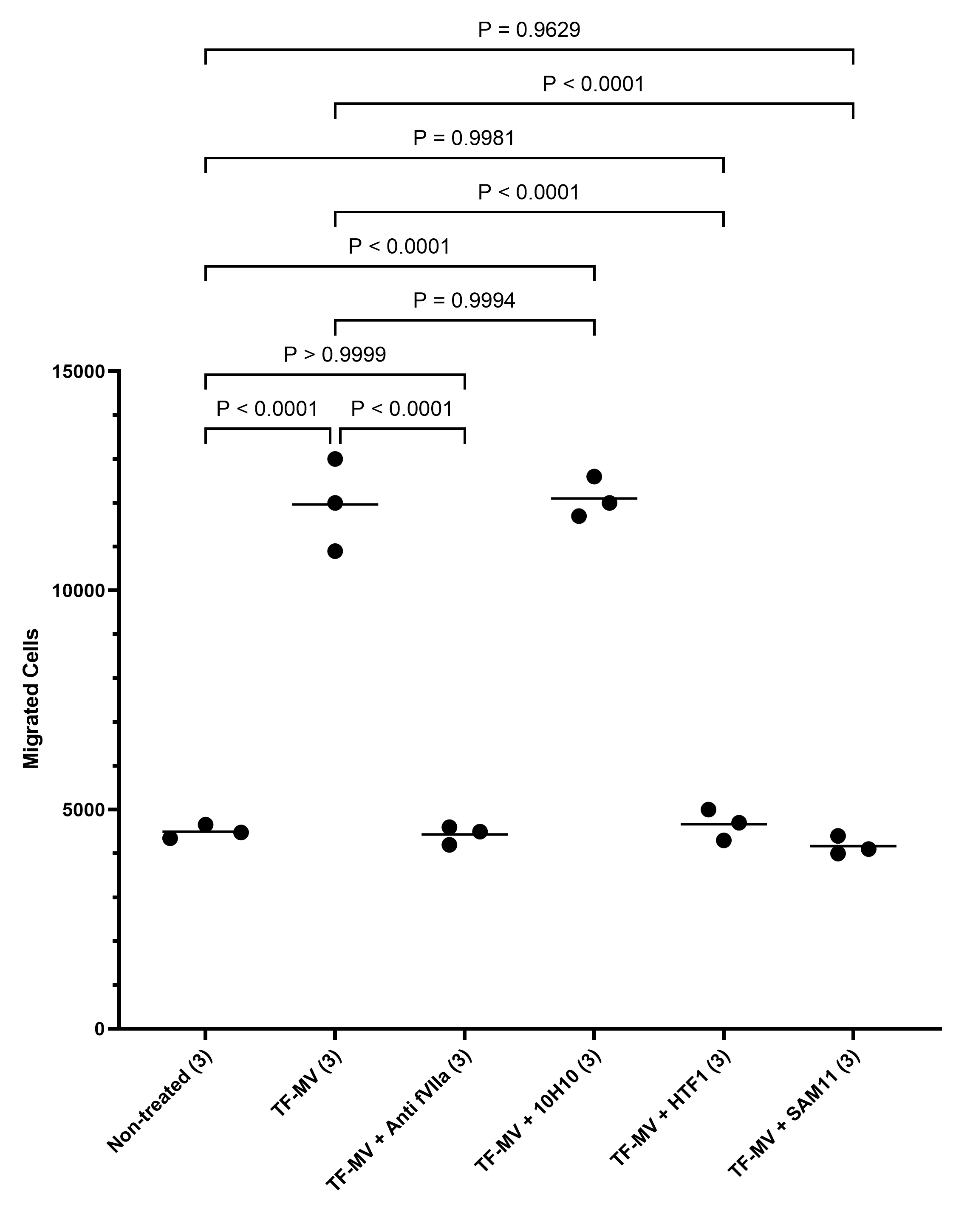


Supplemental Figure 5- MV were prepared from ECV304 (5 × 10^5^) transfected to express TF_Wt_-tGFP and pre-incubated with mouse anti-human-TF antibodies, 10H10 (20 µg/ml) or HTF1 (20 µg/ml), or an inhibitory polyclonal rabbit anti-human fVIIa antibody (10 µg/ml). Additionally, HCASMC were incubated with an inhibitory mouse anti-human PAR2 antibody, SAM11 (20 µg/ml). HCASMC (3 × 10^4^) were stimulated with MV at 37˚C for 18 h. The cells were then fixed, washed with PBS and the cells on the upper side of the chamber were then scraped off. The cells were stained with crystal violet and the numbers of migrating cells were determined by eluting the crystal violet and measuring the absorptions at 595 nm. Presented data include the calculated mean values from 3 biological repeats. Statistical analysis was carried out using the GraphPad Prism version 9.0 and significance was determined using one-way ANOVA (analysis of variance) and Tukey’s honesty significance test.

A)


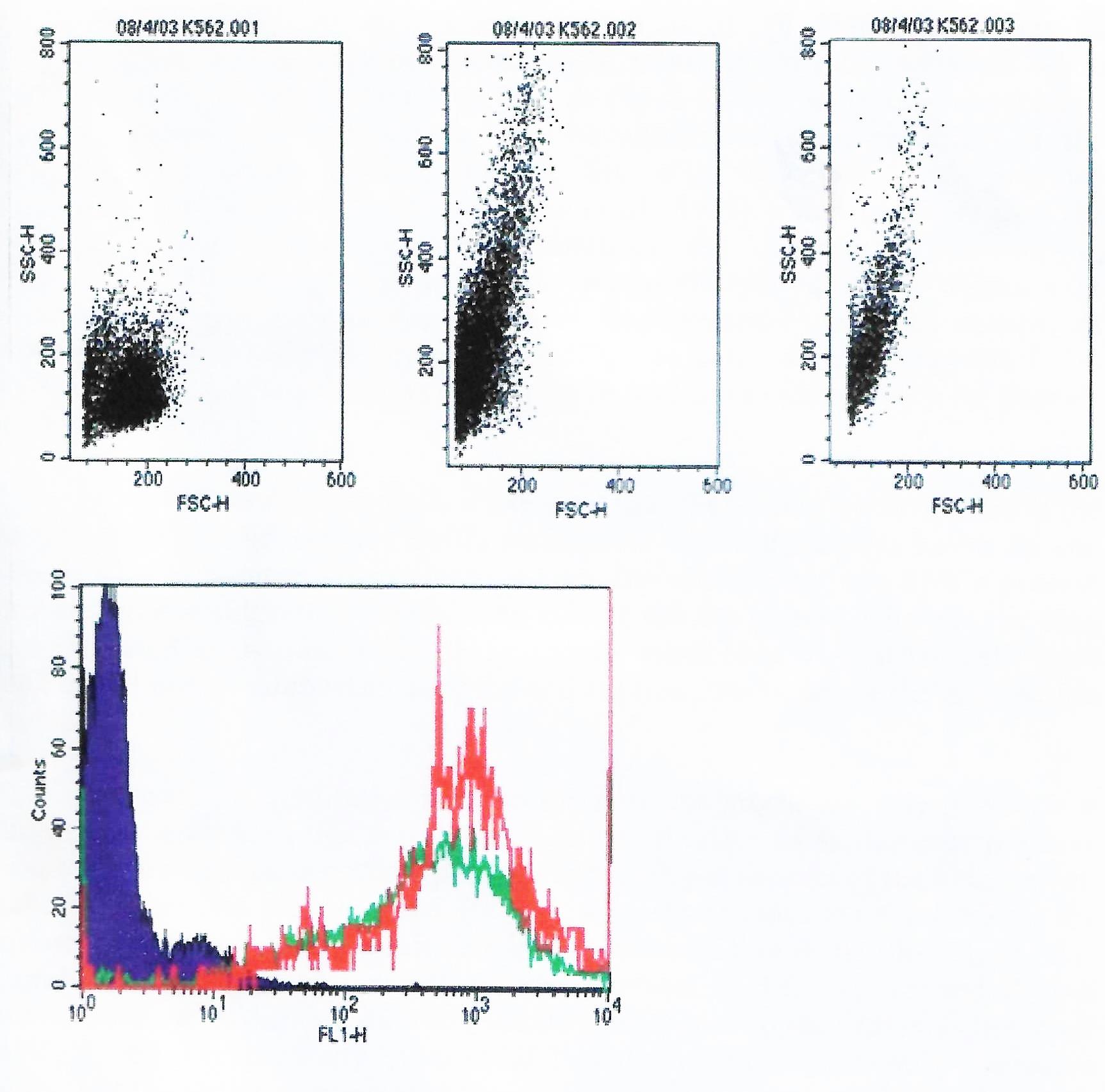


Non-transfected Transfected Transfected

before wash after PBS wash

B)

Supplemental Figure 6- A) Flow cytometric determination of the efficiency of peptide transfection into HCASMC. Cells were transfected with a peptide corresponding to the C-terminal of human tissue factor-FITC conjugated (500 ng) with Chariot reagent, in order to establish the transfection efficiency against control cells. B) Cells were analysed by flow cytometry before (red) and after washing with PBS (green) against non-transfected cells (blue area). The data is representative of three independent experiments.


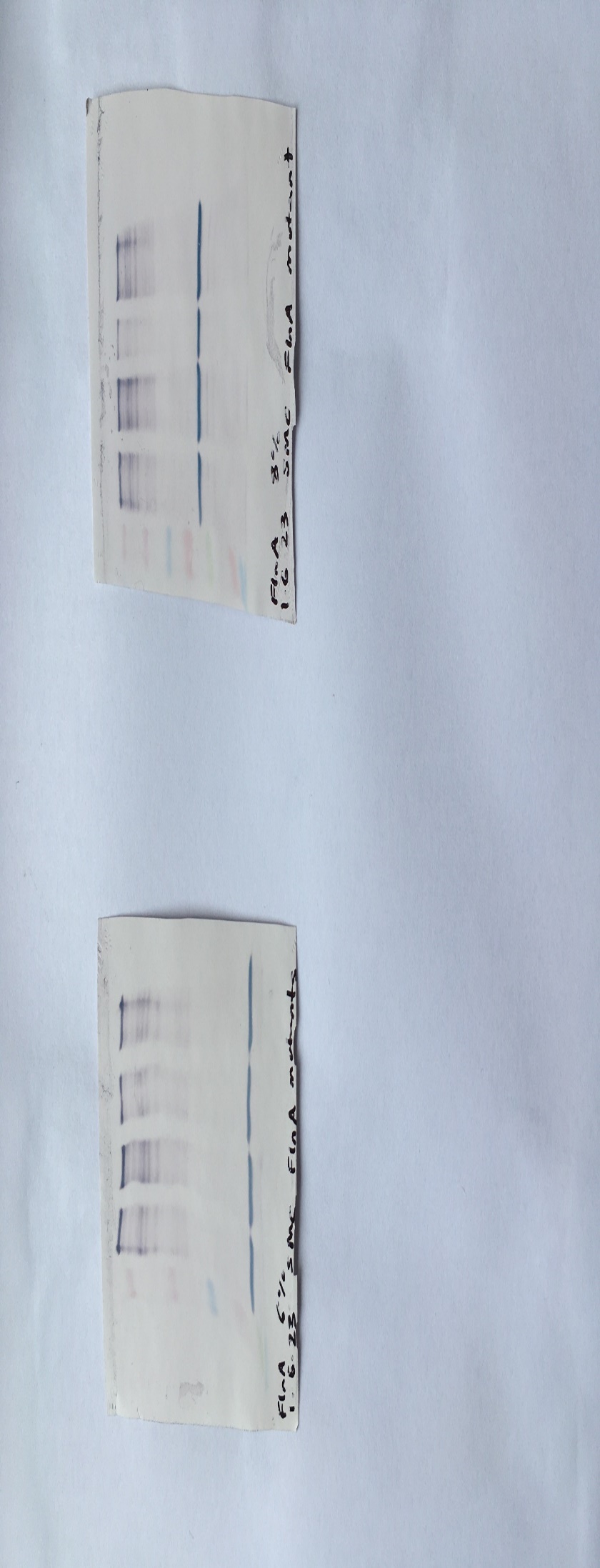

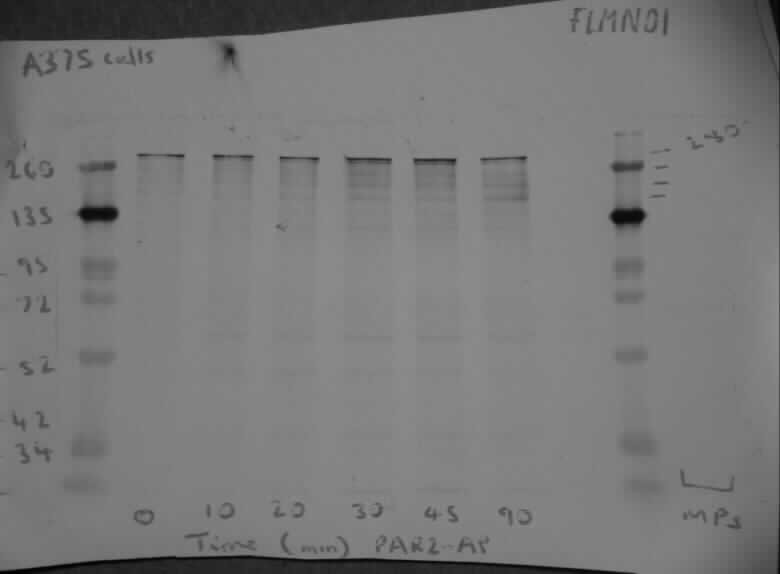
A) B)

Filamin-A

260 kDa

140 kDa

100 kDa

70 kDa

Filamin-A

260 kDa

140 kDa

100 kDa

70 kDa

Δ22-24 Δ23-24 Δ4 Non-transfected Marker marker Δ22-24 Δ23-24 Δ24 Non-transfected

Supplemental Figure 7- HCASMC were transfected with the pcDNA 3 c-myc constructs to express the wild type or mutant forms of filamin-A, lacking repeats domains (22-24, 23-24 and 24 alone). The cells were permitted to express the proteins for 48h, lysed and examined by western blot analysis. A) The expression of the recombinant proteins was confirmed using a biotin-conjugated mouse anti-c-myc antibody (diluted 1:3000 v:v), probed with HRP-conjugated streptavidin (diluted 1:4000 v:v) and developed using TMB stabilized substrate. B) Parallel sets of samples were also examined using a rabbit anti-filamin-A antibody (EP2405Y) (diluted 1:3000 v:v), probed with alkaline phosphatase-conjugated goat anti-rabbit antibody (diluted 1:4000 v:v) and developed using Western Blue substrate. The micrographs are representative of three independent experiments
